# Blocking of amino acid transporter *OsAAP7* promoted tillering and yield by determining basic and neutral amino acids accumulation in rice

**DOI:** 10.1186/s12870-024-05159-5

**Published:** 2024-05-23

**Authors:** Feng Jin, Pengfei Xie, Zhenghan Li, Bowen Wu, Weiting Huang, Zhongming Fang

**Affiliations:** 1 Institute of Rice Industry Technology Research, Key Laboratory of Functional Agriculture of Guizhou Provincial, Department of Education, Key Laboratory of Molecular Breeding for Grain and oil Crops in Guizhou Province, College of Agricultural Sciences, Guizhou University, Guiyang, 550025 China; 2 Key Laboratory of Plant Resource Conservation and Germplasm Innovation in Mountainous Region (Ministry of Education), Guizhou University, Guiyang, 550025 China

**Keywords:** Amino acid, Transporter, Tillering, Yield, Rice

## Abstract

**Background:**

Amino acids are not only the main form of N in rice, but also are vital for its growth and development. These processes are facilitated by amino acid transporters within the plant. Despite their significance, only a few AAP amino acid transporters have been reported.

**Results:**

In this study, we observed that there were differences in the expression of amino acid transporter *OsAAP7* among 521 wild cultivated rice varieties, and it directly negatively correlated with tillering and grain yield per plant. We revealed that OsAAP7 protein was localized to the endoplasmic reticulum and had absorption and transport affinity for amino acids such as phenylalanine (Phe), lysine (Lys), leucine (Leu), and arginine (Arg) using subcellular localization, yeast substrate testing, fluorescent amino acid uptake, and amino acid content determination. Further hydroponic studies showed that exogenous application of amino acids Phe, Lys and Arg inhibited the growth of axillary buds in the overexpression lines, and promoted the elongation of axillary buds in the mutant lines. Finally, RNA-seq analysis showed that the expression patterns of genes related to nitrogen, auxin and cytokinin pathways were changed in axillary buds of *OsAAP7* transgenic plants.

**Conclusions:**

This study revealed the gene function of *OsAAP7*, and found that blocking of amino acid transporter *OsAAP7* with CRISPR/Cas9 technology promoted tillering and yield by determining basic and neutral amino acids accumulation in rice.

**Supplementary Information:**

The online version contains supplementary material available at 10.1186/s12870-024-05159-5.

## Introduction

*Oryza sativa* (Rice) is the primary grain crop globally, particularly in Asia [1]. The swiftly increasing population will be met with a rise in the demand for rice production, making it crucial to enhance rice grain yield to address this future challenge. The grain yield potential of rice is influenced by various components, with tillering being a key factor [2]. In rice plants, tillers originate from basal internodes, and each tiller is crowned with a panicle [3]. Furthermore, tillering influences the panicle number per plant, ultimately regulating grain yield [4].

Nitrogen (N) is a vital nutrient for crop growth and can regulate the process of rice tillering [5, 6]. Plants can uptake both inorganic nitrogen (nitrate and ammonium) and organic nitrogen (amino acids) from the soil. Subsequently, a significant portion of nitrate and a fraction of ammonium undergo assimilation into amino acids. Additionally, amino acids serve as the primary N form for transportation and allocation in rice [7]. Therefore, it is crucial to investigate whether and how amino acids affect rice growth, particularly for tillering.

Amino acid transport in plants is facilitated by amino acid transporters (AATs), and these AATs are divided into two superfamilies: APC (Amino acid, polyamine and choline transporters) superfamily and AAAP (Amino acid/auxin permease) superfamily. Among them, the APC superfamily is divided into three subfamilies: Cationic amino acid transporters (CATs), Amino acid/choline transporters (ACTs) and Polyamine H^+^ symporters (PHSs); the AAAP superfamily is divided into six subfamilies: Amino acid permeases (AAPs), Lysine-histidine-like transporters (LHTs), Proline transporters (ProTs), γ-aminobutyric acid transporters (GATs), Auxin transporters (AUXs) and Aromatic and neutral amino acid transporters (ANTs) [8].

Amino acid permease (AAP) is a type of amino acid transporter capable of absorbing and transporting amino acids [9]. To date, many AAPs have been functionally studied in higher plants. In Arabidopsis, AtAAP1 primarily uptakes glutamate (Glu), histidine (His), and phenylalanine (Phe); the mutant *aap1* can tolerate high levels of amino acids to avoid the suppression of plant growth [10]. Moreover, it has been demonstrated that *AtAAP1* is essential for the synthesis of storage protein and formation of seed yield [11]. *AtAAP2* is involved in the xylem-phloem transfer of amino acids. Interestingly, the *aap2* mutant exhibits improved characteristics in terms of branch number, silique number, seed yield, and nitrogen use efficiency (NUE) compared to those of the wild-type [12, 13]. *AtAAP3* is mainly expressed in the phloem [14]. The *AtAAP6* mutant has a larger rosette width, number of cauline leaves, and seed size compare with the wild-type [15]. Additionally, *AtAAP8* plays an important role in seed development and influences seed number [16].

In rice, 19 AAPs have been identified [17]. Among these genes, OsAAP1 facilitates the transport of neutral amino acids and positively regulates plant growth and yield [18]. Besides, *OsAAP3* negatively controls bud outgrowth, tiller number and grain yield, and reducing *OsAAP3* expression can enhance NUE in rice [19]. Overexpression of *OsAAP4* increases tiller number and grain yield by transporting valine (Val), proline (Pro), threonine (Thr) and leucine (Leu) [20]. *OsAAP5* negatively affects bud outgrowth through cytokinin content mediation [21]. *OsAAP6* regulates the uptake and distribution of amino acids in rice [22]. Amino acid transporter *OsAAP14* determines bud outgrowth under melatonin treatment, thereby influencing rice tillering and grain yield [23]. Overexpressing of *OsAAP15* in rice enhances both primary and secondary branches in the panicle, attributed to its impact on the concentrations of amino acids Tyr and Val in the rice panicle [24]. In maize, *ZmAAP6* positively modulates grain protein [25].

Gene editing technology encompasses the methodology aimed at modifying specific sites within a target gene sequence [26]. CRISPR/Cas9 technology represents one of the most recent advancements in gene editing with widespread applications in elucidating gene functions across various organisms compared to ZFN and TALEN technologies [27]. The CRISPR/Cas9 gene editing process typically involves two primary steps. Firstly, a specific guide RNA (sgRNA) is employed to target the gene sequence of interest. This sgRNA is synthesized in vitro, utilizing a design tailored to the specific DNA sequence being targeted. Secondly, once the sgRNA has successfully bound to its complementary sequence within the target DNA, the Cas9 protein, which acts as a molecular scissors, facilitates the editing process. Cas9, guided by the sgRNA, precisely cuts the DNA at the intended location, thereby initiating the editing process. In essence, the CRISPR/Cas9 system utilizes the sgRNA to locate the target sequence through base pairing, enabling Cas9 to perform the precise DNA editing [28]. This approach has revolutionized the field of genetic engineering due to its simplicity, efficiency, and versatility.

For RNA-seq, it has evolved alongside second-generation high-throughput sequencing (NGS) [29]. The transcriptome comprises all transcripts produced under specific temporal and spatial conditions, encompassing mRNA and non-coding RNA. It serves as the bridge between genetic information and the biological functions of the proteome [30]. RNA-seq technology not only enables the study of gene function and structural characteristics at the transcriptional level but also unveils the molecular mechanisms involved in biological processes under specific conditions [31, 32]. Presently, the primary experimental process of high-throughput RNA-Seq sequencing technology includes total RNA extraction from samples, obtaining high-quality mRNA, and synthesizing cDNA using mRNA as a template, followed by a series of purification, elution, repair, recovery, and PCR reaction to construct the entire sample library, quality detection through instruments, and qRT-PCR. Finally, the qualified library undergoes sequencing, after which the sequencing data are analyzed to complete the entire technical process.

In this study, our aim was investigation of the function of *OsAAP7* AAPs using CRISPR/Cas9 and RNA-seq technology to elucidate the physiological mechanism of *OsAAP7* on amino acid absorption and transport in rice. Our findings have significant implication for rice high-yield breeding program with gene editing technology.

## Material and method

### Sequence variation of *OsAAP7* promoter

SNP variants in the promoter of *OsAAP7* (LOC_Os05g34980) were extracted from 521 wild rice varieties worldwide, as provided by RiceVarMap 2.0 (website: http://ricevarmap.ncpgr.cn/) [33]. The 521 wild rice varieties were cultivated in experimental paddy fields in Huaxi District, Guizhou Province, spanning from May to October. The applied fertilizer rate was 270 kg·ha^− 1^, with a nitrogen, phosphorus and potassium ratio of 19%, 7% and 14%, respectively. Three haplotypes (Hap1-Hap3) of *OsAAP7* were identified, and the expression of *OsAAP7* corresponding to Hap1-Hap2 was determined using real-time fluorescence quantitative PCR (qRT-PCR). Primers were designed with Primer Premier 5 and were provided in Supplemental Table S1. Subsequently, associations between the two haplotypes and agronomic traits were examined.

### Generation of *OsAAP7* plant vector

To generate an overexpression vector of *OsAAP7*, the cDNA (1491 bp) of *OsAAP7* was fused to pCAMBIA1306 vector under *35 S* promoter with *Bam*H I and *Kpn* I, and introduced into wild-type ZH11. Due to the SNP mutation sites on exon 4 of *OsAAP7*, we employed double targets to knockout the fourth exon of the *OsAAP7* sequence in ZH11 to improve knockout efficiency with CRISPR/Cas9 technology. For the construction of the *OsAAP7* promoter-GUS vector, a promoter (2113 bp) fragment upstream of the *OsAAP7* coding region was amplified and inserted into pCAMBIA1391Z vector upstream of β-glucuronidase (GUS) gene, utilizing *Bam*H I and *Eco*R I enzymes. All vectors were subsequently transformed into *Agrobacterium*, and transgenic lines were generated by infecting calli from japonica rice ZH11 with *Agrobacterium* [34]. The T2-generation was selected for subsequent experiments. Primer design was carried out using Primer Premier 5 and SnapGene, with details in Supplemental Table S1.

### Rice growth conditions and agronomic traits analysis

ZH11, *OsAAP7* overexpression (OE) and CRISPR (C) lines were planted in the Huaxi and Lingshui experimental fields of Guizhou University with a fertilizer application of (N/P/K = 19%/7%/14%) 270 kg·ha^− 1^. During the rice maturing stage, 30 plants of each line were counted for tiller number and grain yield. T2-overexpressing transgenic lines of *OsAAP7* were detected by qRT-PCR, and CRISPR lines of *OsAAP7* were identified using sequencing. All primers were designed using Primer Premier 5 with details in Supplemental Table S1.

### Subcellular localization

The cDNA sequence of *OsAAP7* was amplified and fused with the GFP (Green Fluorescent Protein) of HBT plasmid. Subsequently, the recombinant vector was transiently transformed into rice protoplasts. *AtWAK2*, which is located on the endoplasmic reticulum membrane, was used with mCherry as a membrane localization marker. Fluorescence was detected with confocal microscope (Nikon, Japan). The information of all primers was designed by Primer Premier 5 with details in Supplemental Table S1.

### GUS staining and paraffin-slicing analysis

To obtain *OsAAP7* expression pattern, GUS staining of different tissues of *OsAAP7* promoter-GUS transgenic lines was executed with GUS Blue Kit (Huayueyang, China). GUS staining was performed according to a previously described histochemical staining method [5]. Then, the photos were taken by tissues with stereomicroscope (Olympus, Japan). For paraffin-slicing analysis, the solution of 50% ethanol: 10% formaldehyde: 5% acetic acid was added in the tissues, then water in gradient 50–100% ethanol was reduced, and finally it was buried in paraffin. These samples were sectioned with slicing instrument (Leica, Germany) and were examined with microscope (Zeiss, Germany).

### Yeast complementation assays of *OsAAP7*

The empty vector and pDR196-*OsAAP7* construct were transformed into 22Δ10α of yeast mutant (*Saccharomyces cerevisiae*). The wild-type strain 23344c served as a control to monitor yeast growth. Yeast transformation was carried out using the Yeast Transformation Kit (Coolaber, China). Transformants were then incubated at 30 °C for 2–4 d after plating onto screening medium plates (SD-Ura solid medium). Colonies were subsequently grown in YPDA liquid medium until the optical density (OD) at 600 nm (Ab600) reached approximately 0.6-1.0. The yeast culture was then centrifuged at 5000 rpm for 2 min in a 1.5 mL centrifuge tube, then the supernatant was discarded, and the pellet was washed with ddH_2_O 3–4 times. The yeast culture was diluted with ddH_2_O to a specific absorbance value. 7 µL of each yeast solution at different dilutions was placed on uracil-free YNB solid medium (lacking amino acids, NH_4_^+^ and uracil; Coolaber, China), 3 mM (NH_4_)_2_SO_4_ or 3 mM of various amino acids was added alone as the sole N source. The yeasts were then incubated with sterile water at dilutions of 1, 10, 100 and 1000 to achieve OD600 values of 0.1, 0.01, 0.001, and 0.0001, respectively. The plates were inverted and placed in a thermostat at 30 °C. After being inverted and incubated at 30 °C for 2–3 d, they were observed with camera.

### Total N and amino acid determination

Total N concentration was determined with a total nitrogen analyzer (SKD-100, Peiou, China). N utilization efficiency (NUtE, %) was calculated with equation: [Grain yield (g) / (Grain N concentration (g) + Straw N concentration (g))] × 100. The concentrations of individual free amino acids were measured as follows: 1 g of sample was incubated in 10 mL of 80% ethanol at 80 °C for 20 min, then the supernatant was transferred with repeated steps. The solution was dried to remove ethanol and water at 80 °C, and 1 mL of 0.5 mM NaOH was added. After centrifugation at 14,000 rpm for 20 min, the supernatant was filtered using a 2 μm filter membrane. Finally, the filtrate containing amino acids was analyzed using HPLC Agilent-1260 (Agilent, America).

### Fluorescent amino acid uptake experiment

To delve deeper into amino acid transport, ZH11, *OsAAP7* OE and C lines were subjected to treatment with 1 mM Fluorescent amino acid Arg, Phe or Lys for 2 h, 6 or 10 h. Subsequently, the rice seedlings were examined using a Chemiluminescence Apparatus (Qinxiang, China), and the fluorescent intensity was quantified utilizing Bandscan [23, 24].

### Hydroponic culture and seedling growth observation

For hydroponic culture, rice seedlings of ZH11, *OsAAP7* OE and C lines were initially grown in basic rice nutrient solution composing of 1 mM NH_4_NO_3_, 0.32 mM NaH_2_PO_4_, 0.51 mM K_2_SO_4_, 1.0 mM CaCl_2_, 1.65 mM MgSO_4_, 8.9 µM MnSO_4_, 0.5 µM Na_2_MoO_4_, 18.4 µM H_3_BO_3_, 0.14 µM ZnSO_4_, 0.16 µM CuSO_4_, and 40.0 µM FeSO_4_ for 1 week. Then the seedlings were transferred to a solution supplemented with 1.0 mM NH_4_NO_3_ and one of the amino acids Arg, Phe or Lys. The lengths of both the first and second axillary buds were assessed with stereomicroscope (Olympus, Japan). The rice seedlings were grown in boxes (525 mm×360 mm×230 mm) with the solution in a phytotron under condition of 30 °C for 14 h and 25 °C for 10 h. The culture solution was replaced every 3 d.

### RNA extraction and qRT-PCR

Total RNA from rice tissue was extracted using TRIzol according to the manufacturer’s instructions (Vazyme, China). The isolated total RNA (approximately 3 µg) served as the initial material for first-strand cDNA synthesis, employing M-MLV reverse transcriptase (Vazyme, China). qRT-PCR was carried out in a 10 µL reaction mixture containing 1 µL of cDNA, 1 µL of primers, 5 µL of Mix (Vazyme, China), and 3 µL of ddH_2_O under the following conditions: 95 °C for 2 min (1 cycle); 95 °C for 30 s, 60 °C for 30 s, and 72 °C for 30 s (35 cycles); and 72 °C for 1 min (1 cycle). The sequences of *OsAAP7* cDNA or promoter were amplified in a 20 µL reaction mixture comprising 1 µL of DNA, 10 µL of Mix (Vazyme, China), 1.5 µL of primers and 6 µL of ddH_2_O under the following these conditions: 95 °C for 3 min (1 cycle); 95 °C for 30 s, 50–68 °C for 30 s, and 72 °C for 2 min (30–35 cycles); and 72 °C for 10 min (1 cycle).

### RNA-seq analysis

Axillary buds of sixty seedlings were collected from ZH11, *OsAAP7* OE1 and C1 lines each with a replicate weight of 0.5 g. RNA-seq analysis was conducted by Personalbio (Personalbio, China). The resulting data are accessible at the National Center for Biotechnology Information (NCBI). Clean data were aligned to the rice genome sequence (*Oryza sativa*. IRGSP-1.0) [35]. Reads were counted and summarized using feature counts [36]. Differentially expressed genes (DEGs) were identified utilizing the DESeq package, with a significance thresholds of *p* value < 0.05, and a fold-change > 1.4 set as criteria for significant differential expression.

### Statistical analysis

The statistical charts were created using GraphPad Prism 8, while the heatmaps were generated using TBtools. To assess statistical differences, a t-test was employed with SPSS (IBM, Inc.). Significance levels were denoted as follows: *, **, and *** represented significant differences at *P* < 0.05, *P* < 0.01, and *P* < 0.001, respectively. Additionally, Tukey-Kramer’s multiple range test was conducted using SPSS software, with significant difference indicated at *P* < 0.05. The data are presented as mean ± standard deviation values.

## Results

### Promoter of amino acid transporter *OsAAP7* was divergent between indica and japonica rice

**Fig. 1:**
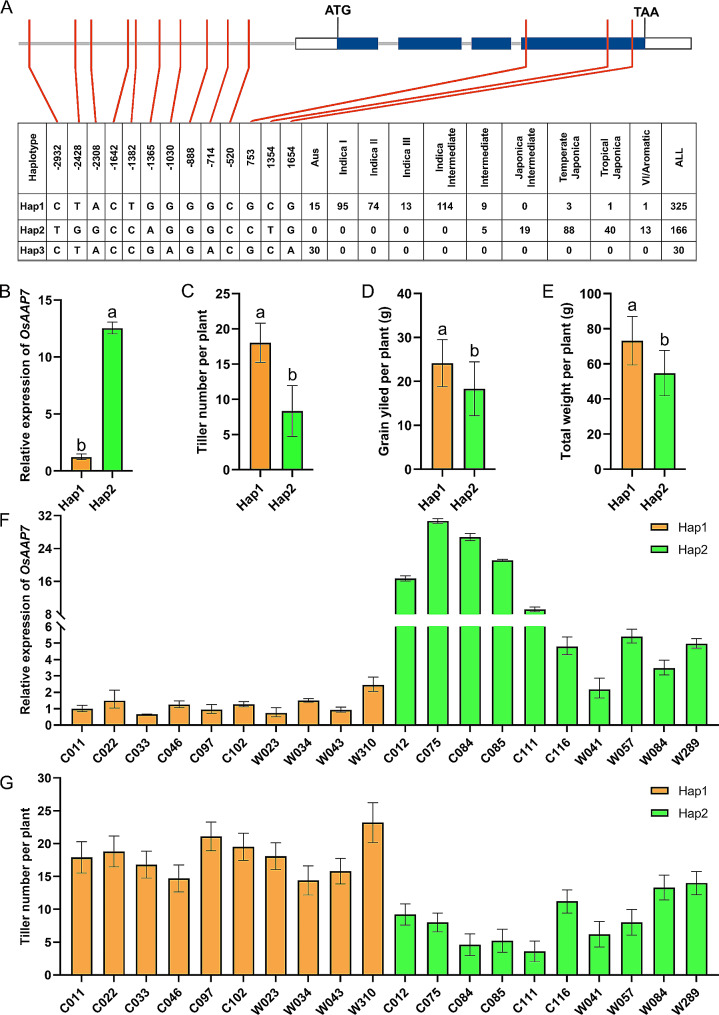
*OsAAP7* was divergent between *indica* and *japonica*. (**A**) SNPs of *OsAAP7* promoter in 521 rice varieties. (**B**) Expression of *OsAAP7* in the basal part of rice seedlings with three haplotypes. (**C**-**E**) Tiller number per plant, grain yield per plant and total weight with every variety of three haplotypes. (**F**) Expression of *OsAAP7* in seedling axillary bud of ten individual varieties between Hap1 and Hap2. (**G**) Tiller number of these varieties between Hap1 and Hap2. Error bars represent the SD (*n* = 4). Various letters represent significant differences at *P* < 0.05. The rice varieties were planted in paddy at Guiyang under fertilizer of (N/P/K = 19%/7%/14%) 270 kg·ha^-1^

Promoter of amino acid transporter *OsAAP7* (2600 bp upstream of the codon ATG) was investigated in 521 wild rice varieties worldwide utilizing haplotype (Hap) analysis with the Rice Variation Map v2.0 [37]. The findings revealed three major Haps among these cultivars (Fig. 1A). Notably, Hap1 predominated in *indica*, whereas Hap2 was prevalent in *japonica* (Fig. 1A). Analyzing the relative expression of *OsAAP7* among the three haplotypes unveiled a distinct expression pattern in the *japonica* haplotype (Hap2) compared to the *indica* haplotype (Hap1; Fig. 1B). Furthermore, it was observed that *indica* haplotype exhibited more tillers than *japonica* (Fig. 1C). The yield of Hap1 surpassed that of Hap2 (Fig. 1D), and the total weight of Hap1 was significantly higher than that of Hap2 (Fig. 1E). Additionally, a comparison of *OsAAP7* expression in randomly selected 10 *indica* and 10 *japonica* wild cultivars among with Hap1 and Hap2, demonstrated lower expression in *indica* with Hap1 than in *japonica* with Hap2 (Fig. 1F). Conversely, *indica* with Hap1 displayed more tillers than *japonica* with Hap2 (Fig. 1G). These results underscored differences in the promoter sequences of *OsAAP7* between *indica* and *japonica*. Consequently, *indica* varieties with Hap1 exhibited increase of tillering but decrease of *OsAAP7* expression compared to *japonica* varieties with Hap2, implying a potential correlation between elevated *OsAAP7* expression and reduced rice tillering.

### Expression pattern of *OsAAP7* at different stages in rice tissues, and its subcellular localization

**Fig. 2:**
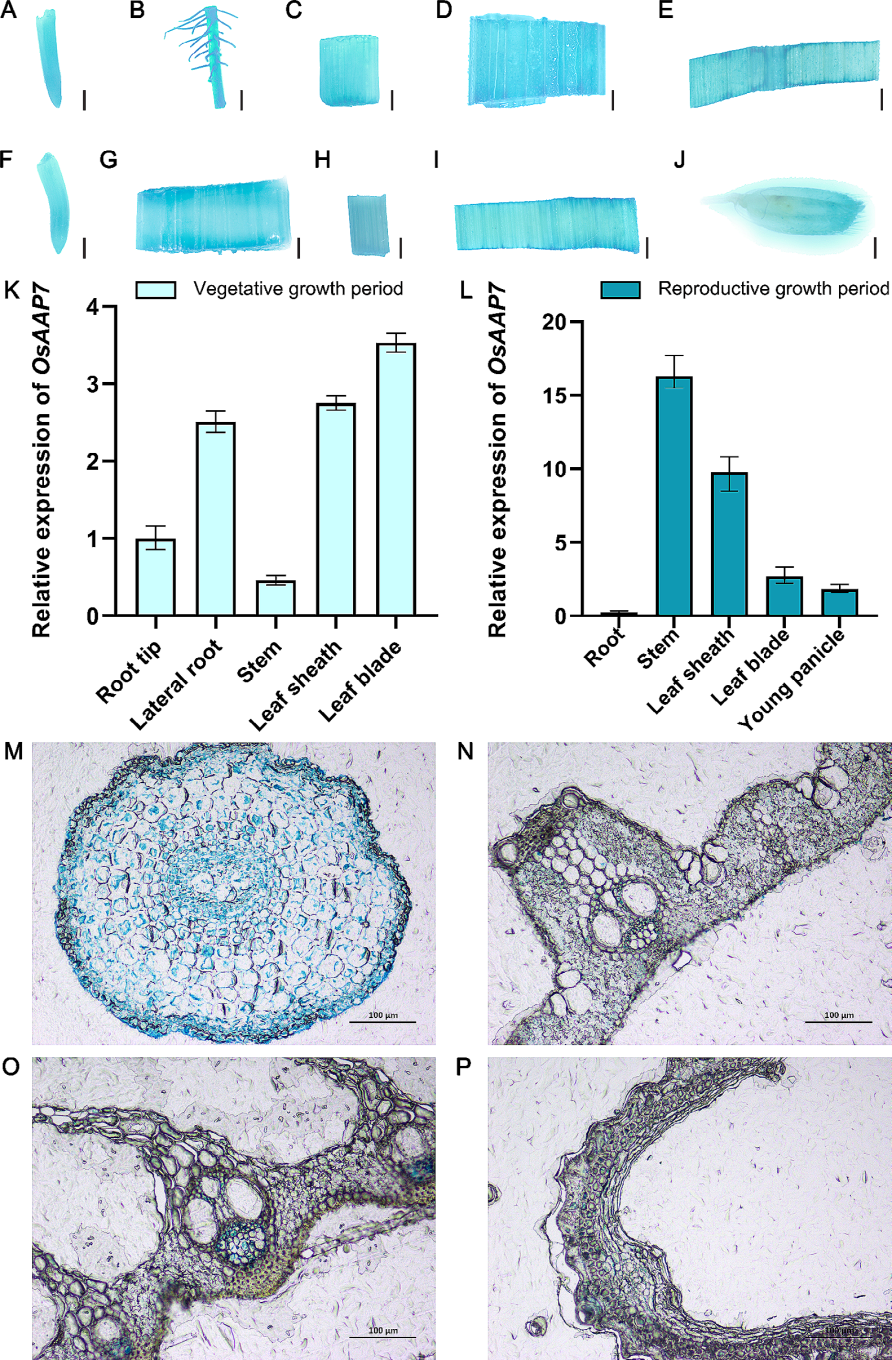
Staining and section analysis of *OsAAP7* promoter-GUS transgenic lines. (**A**-**E**) *OsAAP7* promoter-GUS staining was performed with root tip, lateral root, stem, leaf sheath and leaf blade of *pOsAAP7*-GUS transgenic plants during vegetative stage. Scale bars represent 1 mm. (**F**-**J**) *OsAAP7* promoter-GUS staining was performed with root, stem, leaf sheath, leaf blade and young panicle of *pOsAAP7*-GUS transgenic plants during reproductive stage. Scale bars represent 1 mm. (**K**-**L**) Relative expression of *OsAAP7* in various tissues of ZH11 was detected during vegetative and reproductive stages. (**M**-**P**) Paraffin sections of the stained root, leaf blade, leaf sheath and panicle. Scale bars represent 100 μm. Error bars represent the SD (*n* = 4)

To identify the expression pattern of *OsAAP7*, p*OsAAP7*-GUS transgenic lines were established, and various tissues both during vegetative and reproductive stages were subjected to GUS staining. The GUS-staining experiments revealed that *OsAAP7* expression was elevated in lateral roots, leaf sheaths and leaf blades (Fig. 2B, D, E) during vegetative stage but was diminished in root tips (Fig. 2A) and stems (Fig. 2C). Conversely, during the reproductive stage, *OsAAP7* expression heightened in stems (Fig. 2G) and leaf sheaths (Fig. 2H) but declined in roots (Fig. 2F), leaf blades (Fig. 2I), and young panicles (Fig. 2J). qRT-PCR analysis further corroborated the expression profiles of *OsAAP7* during the vegetative stage (Fig. 2K) and reproductive stage (Fig. 2L). The vascular tissues and parenchyma cells of leaf blades (Fig. 2N) and leaf sheaths (Fig. 2O) exhibited higher GUS activity during the reproductive stage, whereas roots (Fig. 2M) and young panicles (Fig. 2P) displayed lower GUS activity.

Besides, it was revealed that the expression of *OsAAP7* in lateral roots was elevated after a 2 h treatment with basic amino acids by GUS staining. Furthermore, a higher expression of *OsAAP7* in lateral roots was observed by 8 h treatment with acidic or neutral amino acids (Supplementary Fig. S1A). Subsequent qRT-PCR analysis further confirmed the expression profile of *OsAAP7* under treatment with basic, acidic and neutral amino acids (Supplementary Fig. S1B).

Furthermore, the study demonstrated that the GFP control exhibited fluorescent proteins distributing in both the plasma membrane and nucleus within the rice protoplasts (Supplementary Fig. S2A). In contrast, the fluorescence pattern of *OsAAP7*-*GFP* was notably concentrated in the endoplasmic reticulum (ER) membrane, consistenting with the localization of a co-marker which also distributed in the ER membrane. This observation suggests that the OsAAP7 protein is predominantly localized within the endoplasmic reticulum membrane (Supplementary Fig. S2B).

### Substrate transport of OsAAP7 protein with yeast complementation assay and fluorescent amino acid uptake experiment

**Fig. 3:**
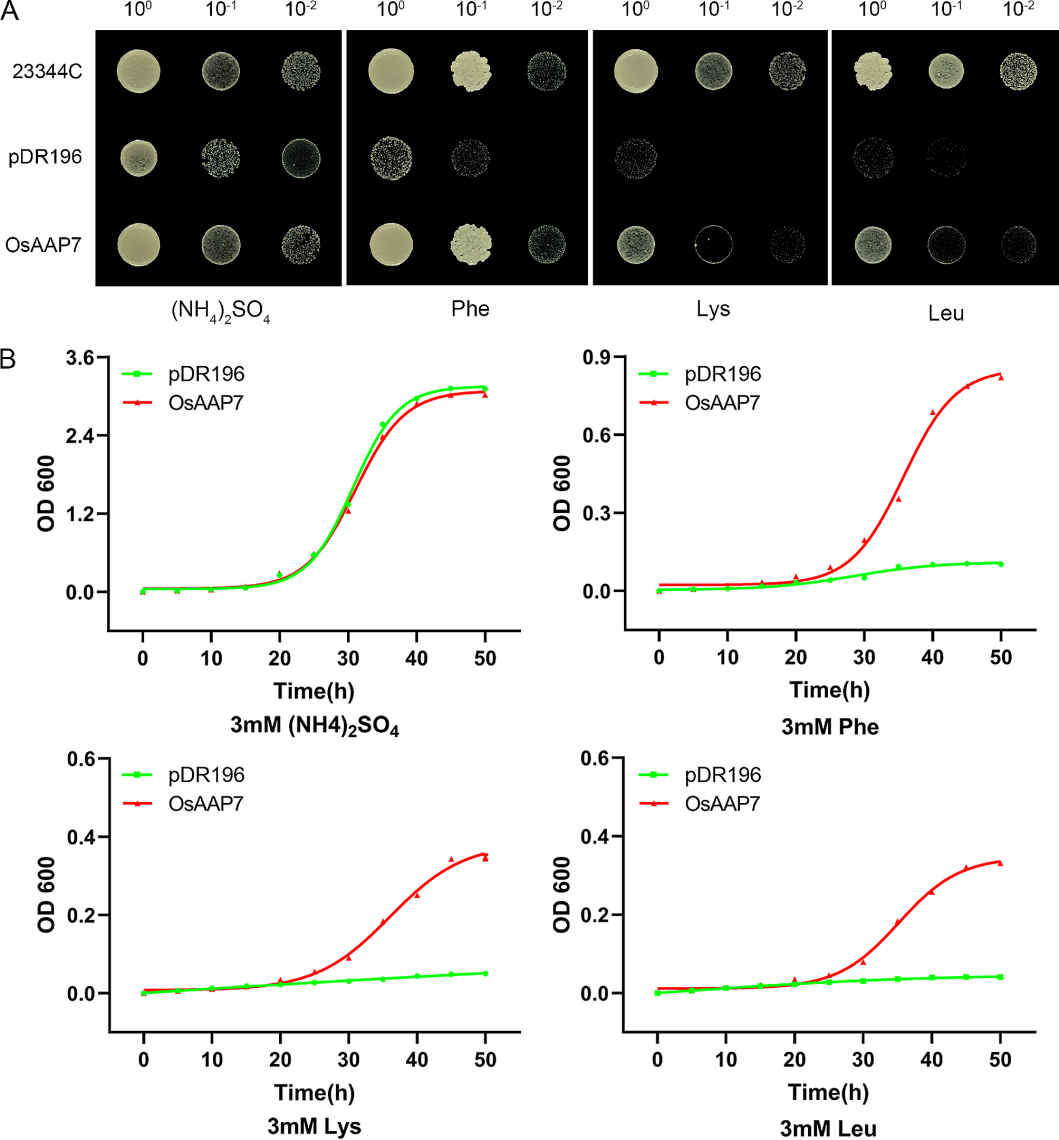
Yeast growth complementation assay with various amino acids. (**A**) YNB solid medium with 3 mM (NH_4_)_2_SO_4_ or amino acid as sole nitrogen source. The pDR196 (empty vector) was used with negative control. Photos were taken after 72 h of growth under 30 °C. (**B**) Growth rates were measured using yeast mutants with *OsAAP7*-pDR196 or empty vector pDR196 in (NH_4_)_2_SO_4_, phenylalanine, lysine or leucine. Yeast were cultured in liquid media with 3 mM amino acids or 3 mM (NH_4_)_2_SO_4_ as sole N source for 50 h. OD600 was detected every 5 h. Error bars represent SD (*n* = 3)

To ascertain OsAAP7 protein’s role in amino acid transport, we examined substrate transport using the mutant yeast strain 22Δ10α. Medium containing 3 mM amino acids Phe, Lys or Leu was crucial for the growth of 22Δ10α-transformed OsAAP7 protein (controlled by pDR196), respectively (Fig. 3A). To assess OsAAP7 protein is transport properties, yeast cells expressing the transporter were cultured in liquid culture with 3 mM Phe, Lys or Leu as the sole N source (Fig. 3B). Results indicated that the growth rate of cells expressing *OsAAP7* in pDR196 was faster than those of empty pDR196 vector for these amino acids, monitoring with absorbance of 600 nm light (Fig. 3B). Previous substrate testing also revealed OsAAP7 protein had ability to transport amino acid Arg [38]. These findings suggest that OsAAP7 protein predominantly transports amino acids Phe, Lys, Leu and Arg.

### *OsAAP7* negatively regulated grain yield in rice

To investigate the impact of *OsAAP7* expression changes on rice growth, we generated *OsAAP7* overexpression (OE) lines under the 35 S promoter and *OsAAP7* mutant (C) lines using CRISPR/Cas9 technology. We obtained 24 T0 generation seedlings of OE and 24 T0 generation seedlings of C lines with via the *Agrobacterium*-mediated method. Sequencing of these C lines mutants revealed a total of 11 plants with 860 bp deletion, 2 bp deletion and 1 bp insertion mutation (Supplementary Fig. S3A, S4). It resulted in transformation of 19 mutant lines with verification of the Cas9 protein-encoding gene (Supplementary Fig. S3B). Subsequently, seven mutant lines from the T0 generation were selected and propagated to the T1 generation, and three mutant lines were confirmed through sequencing (Supplementary Fig. S3C, S5). The absence of the Cas9 protein was confirmed in the T2 generation (Supplementary Fig. S3D).

**Fig. 4:**
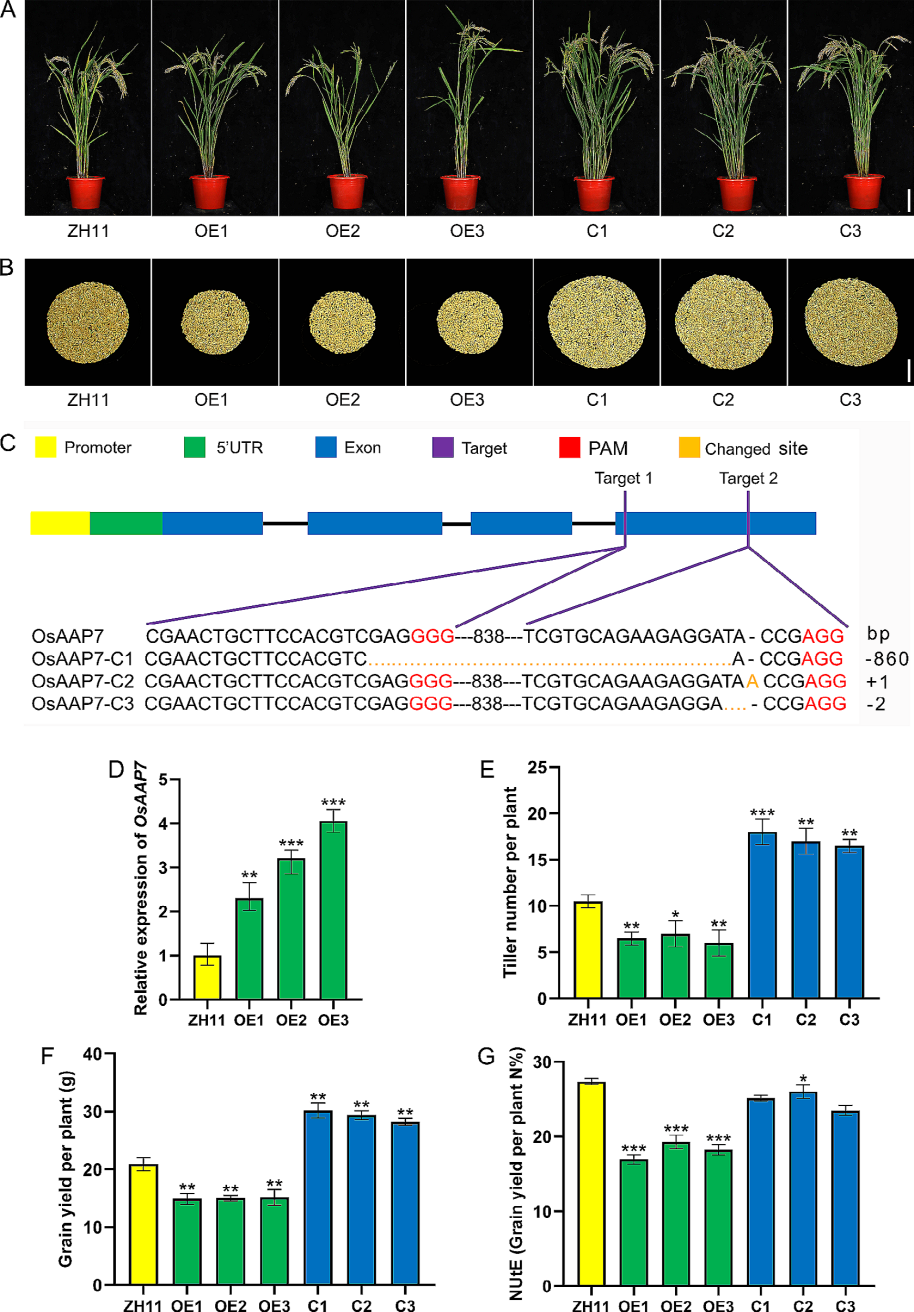
Phenotypes of *OsAAP7* transgenic lines planted in paddy. (**A**) The maturity performance of wild-type ZH11, *OsAAP7-*overexpressing (OE1-3) and CRISPR (C1-3) lines. Scale bars represent 15 cm. (**B**) Grain yield per plant of ZH11, *OsAAP7* OE1-3 and C1-3 lines. Scale bars represent 2 cm. (**C**) Sequencing of *OsAAP7* of T2 generation C1-C3 lines (from T0-9, T0-12 and T0-16). (**D**) Relative expression of *OsAAP7* of T2 generation in ZH11 and OE1-3 lines (from T0-11, T0-14 and T0-22). (**E**-**G**) Tiller number per plant, grain yield per plant and NUtE of ZH11, *OsAAP7* OE1-3 and C1-3 lines (Three lines were selected for OE and C respectively, and 20 samples were repeated for each line). Error bars represent the SD. *, ** and *** represent significant differences at *P* < 0.05, *P* < 0.01 and *P* < 0.001, respectively

The same procedure was followed to verify the hygromycin gene in 24 T0 generation OE lines, leading to the successful transformation of 12 lines (Supplementary Fig. S3E). The expression of the *OsAAP7* gene in these 24 lines is shown in Supplementary Fig. S6. Among these, 6 lines with the higher expression were selected and propagated to the T1 generation to confirm *OsAAP7* expression and the presence of the hygromycin gene (Supplementary Fig. S3F, S7). The expression of *OsAAP7* was verified in OE lines, and the sequence of *OsAAP7* was confirmed in C lines, respectively in T2 generation (Fig. 4C, D). Finally, these three different C lines (C1-C3 derived from T0-9, T0-12, and T0-16) and three OE lines (OE1-OE3 derived from T0-11, T0-14, and T0-22) in T2 generation were chosen for subsequent experiments (Supplementary Fig. S3G, S8).

To assess the impact of altered *OsAAP7* expression on traits, we determined tiller number and grain yield per plant of OE and C lines in paddy field. We observed a reduction in tiller number per plant in OE lines compared to the wild-type, whereas an increase was noted in C lines (Fig. 4A, E). Additionally, in comparison with ZH11, the grain yield per plant decreased in OE lines but increased in C lines, with the grain yield per plant of C lines significantly surpassing that of OE lines (Fig. 4B, F). Furthermore, CRISPR/Cas9-mediated *OsAAP7* knockout mutants exhibited enhanced NUtE compared to ZH11, while NUtE decreased in OE lines (Fig. 4G).

### *OsAAP7* regulated amino acids accumulation of phenylalanine, lysine and arginine in rice

**Fig. 5:**
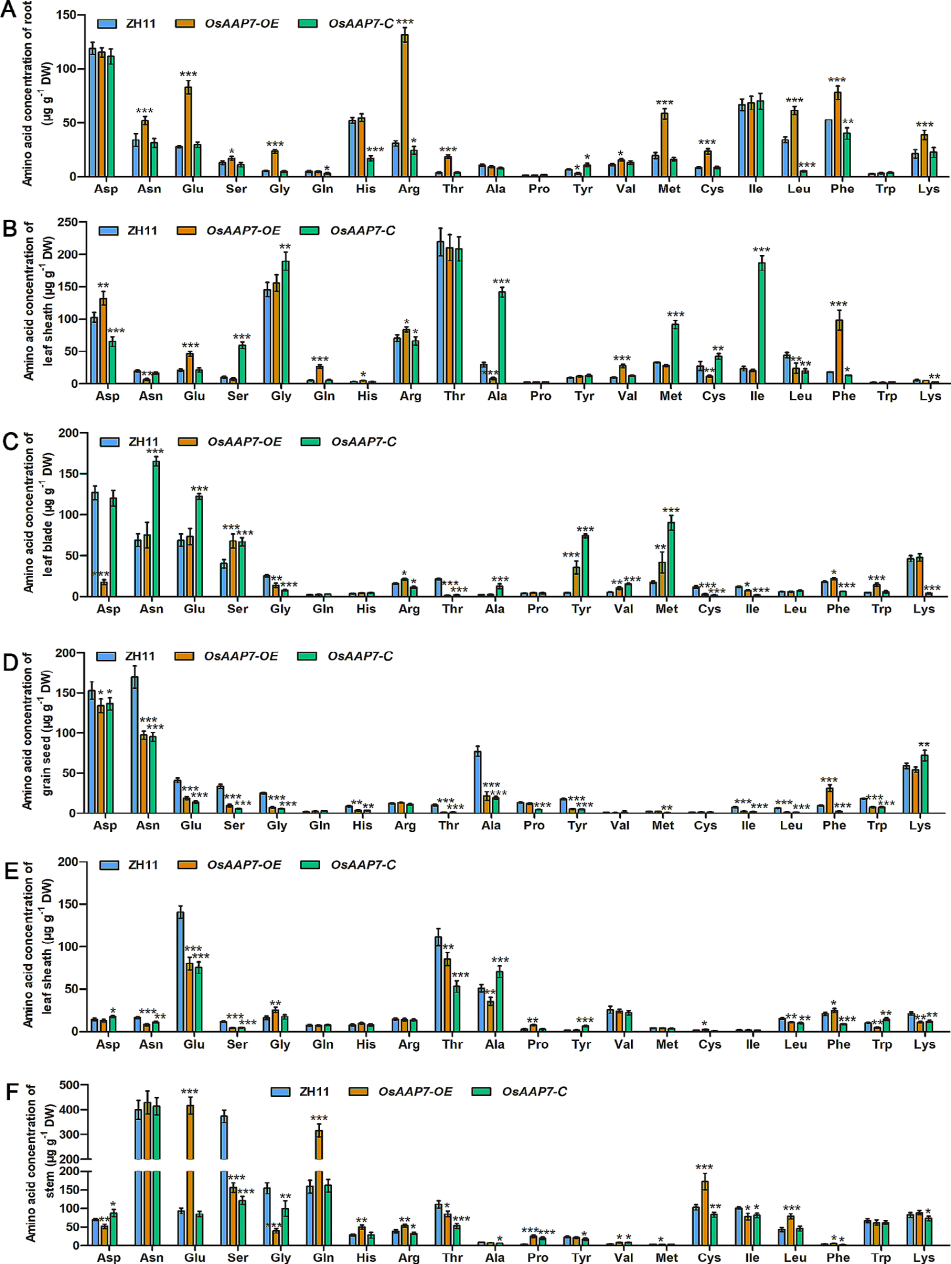
Influence of *OsAAP7* on amino acid concentrations in various rice tissues of control, OE and C lines during vegetative and reproductive stages. (**A**-**C**) Different amino acid concentration of ZH11, *OsAAP7*-overexpressing lines (*OsAAP7*-OE) and *OsAAP7* CRISPR lines (*osaap7*) in roots, leaf sheaths and leaf blades during vegetative stage. (**D**-**F**) Different amino acid concentration of ZH11, *OsAAP7*-overexpressing lines (*OsAAP7*-OE) and *OsAAP7* CRISPR lines (*osaap7*) in grain seed, leaf sheath and leaf blade at reproductive stage. Error bars represent SD (*n* = 20, 20 seedlings were randomly selected from the three OE lines or three C lines respectively) for (**A**-**F**). *, ** and *** represent significant differences at *P* < 0.05, *P* < 0.01 and *P* < 0.001, respectively

To further explore the connection between *OsAAP7* expression and amino acid composition in rice, we determined the concentrations of individual amino acids in the root, leaf blade and leaf sheath of transgenic lines during the vegetative stage, as well as in grain seed, leaf blade and leaf sheath during the reproductive stage. The results showed higher concentrations of Phe, Lys and Arg in OE lines compared to ZH11 (Fig. 5A-C). Conversely, concentrations of these amino acids were reduced in seedlings of C lines compared to ZH11 (Fig. 5A-C). Furthermore, during the reproductive stage, concentrations of Phe, Lys, and Arg in OE lines significantly exceeded with those in ZH11 (Fig. 5D-F), while the opposite trend was observed in C lines (Fig. 5D-F). Thus, our findings suggest a significant accumulation of amino acids Phe, Arg, and Lys in *OsAAP7* OE lines, whereas concentrations decreased in C lines.

To further investigate the transport function of OsAAP7 protein for amino acids such as Phe, Lys, and Arg, transgenic seedlings were subjected to treatments with FITC-labeled Phe, Lys, or Arg for durations of 2 h, 6 h, or 10 h, respectively. The results, as illustrated in Supplementary Figure S9, demonstrated stronger fluorescence signals in *OsAAP7* OE lines when treated with FITC-labeled amino acids compared to the control ZH11. Conversely, fluorescence signals were weaker in *OsAAP7* C lines compared to ZH11 under the same treatments (Supplementary Fig. S9).

These findings strongly suggest that OsAAP7 protein plays a crucial role in the transport of amino acids, specifically Phe, Lys, and Arg in rice. The increased fluorescence in the *OsAAP7* OE lines indicates higher uptake and transport of these amino acids, while the reduced fluorescence in the *OsAAP7* C lines suggests a diminished capacity for amino acid transport compared to the control ZH11. This experimental evidence supports the notion that *OsAAP7* is directly involved in the transport of Phe, Lys, and Arg in rice plants.

### *OsAAP7* mediated outgrowth of axillary buds under different concentrations of amino acids phenylalanine, lysine and arginine

**Fig. 6:**
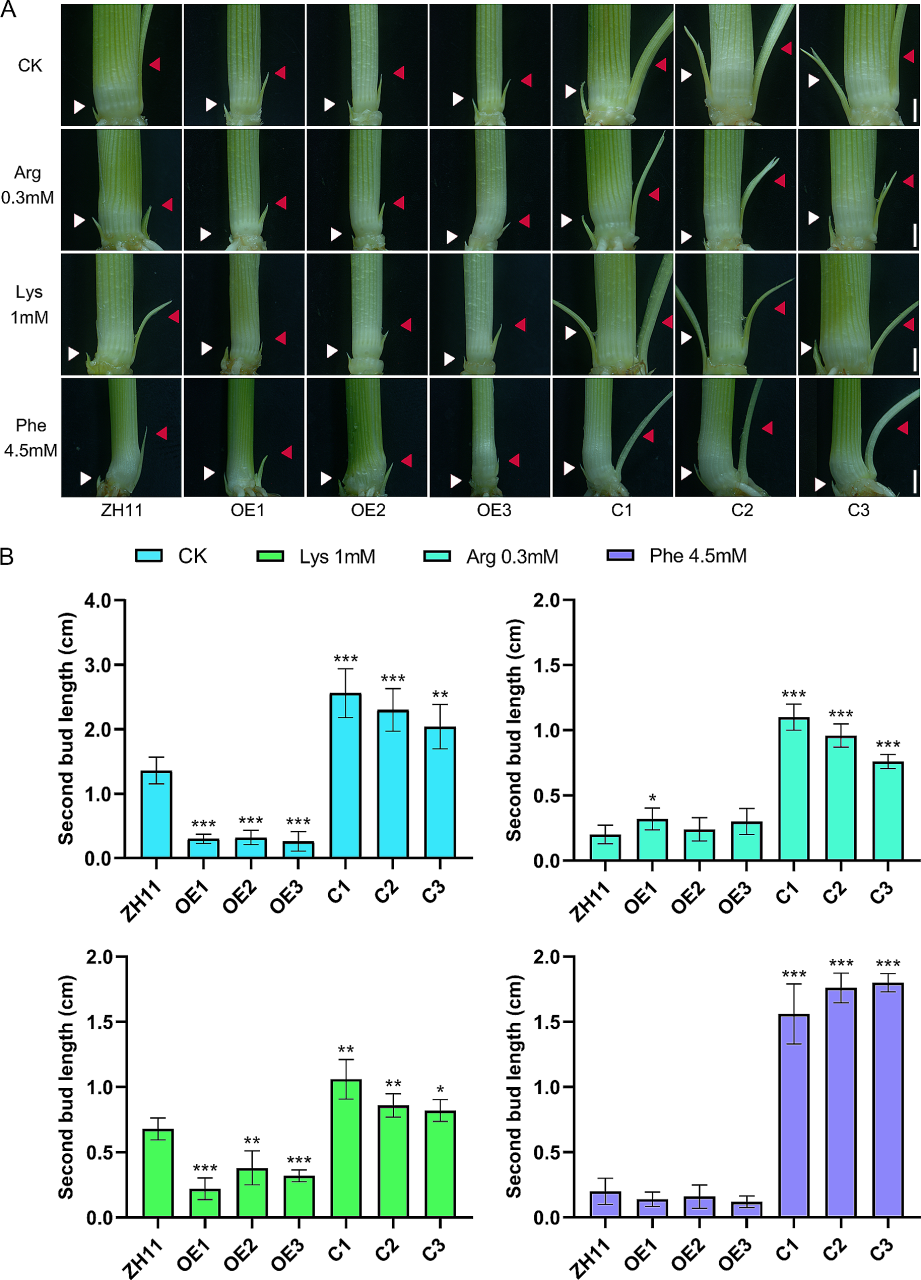
Axillary buds of *OsAAP7* transgenic lines under various amino acid concentrations. (**A**) Phenotypes of axillary buds of wild-type ZH11, *OsAAP7-*overexpressing (OE1-3) and CRISPR (C1-3) lines with 0.3 mM Arg, 1 mM Lys, or 4.5 mM Phe for 35 d. Scale bars represent 2 mm. (**B**) Second bud length of ZH11, *OsAAP7* OE1-3, C1-3 lines with 0.3 mM Arg, 1 mM Lys, or 4.5 mM Phe for 35 days. Error bars represent the SD (*n* = 20). *, ** and *** represent significant differences at *P* < 0.05, *P* < 0.01 and *P* < 0.001, respectively. The white arrow represents the first axillary bud; The red arrow represents the second axillary bud

To investigate *OsAAP7* plays role in rice growth under different Phe, Lys and Arg concentrations, we cultured *OsAAP7* transgenic lines and wild-type ZH11 under various concentrations of these amino acids for 35 d. We found that the plant height and fresh weight of OE lines significantly increased compared to ZH11 under 0.1 mM Arg treatment, while those of C lines remained relatively unchanged. However, OE lines showed a slight decrease in plant height and fresh weight under 0.3 mM Arg treatment, whereas C lines exhibited noticeable increases relative to ZH11 (Fig. 6A, Supplementary Fig. S10). Similar patterns were noted with Phe and Lys treatments (Fig. 6A, Supplementary Fig. S11, S12).

Furthermore, the length of the first bud in control ZH11 and *OsAAP7* transgenic lines remained unchanged significantly under all concentrations of Arg. However, the second buds of OE lines exhibited accelerated growth compared to both control and C lines when treated with 0.1 mM Arg, yet significantly slower growth was observed compared to control and C lines under 0.2 mM and 0.3 mM Arg treatments (Fig. 6B, Supplementary Fig. S13). Similar trends were obtained under Lys and Phe treatments (Fig. 6B, Supplementary Fig. S14, S15). These findings suggest that the elongation of second axillary buds in OE lines was notably enhanced compared to ZH11 and C lines under low exogenous concentration of the amino acids Phe, Lys and Arg. Conversely, the axillary buds growth of OE lines was significantly suppressed compared to ZH11 under high concentrations of these amino acids treatments, while the axillary buds growth of C lines presented an opposite trend.

### *OsAAP7* regulated bud outgrowth by coordinating N, auxin and cytokinin pathways

**Fig. 7:**
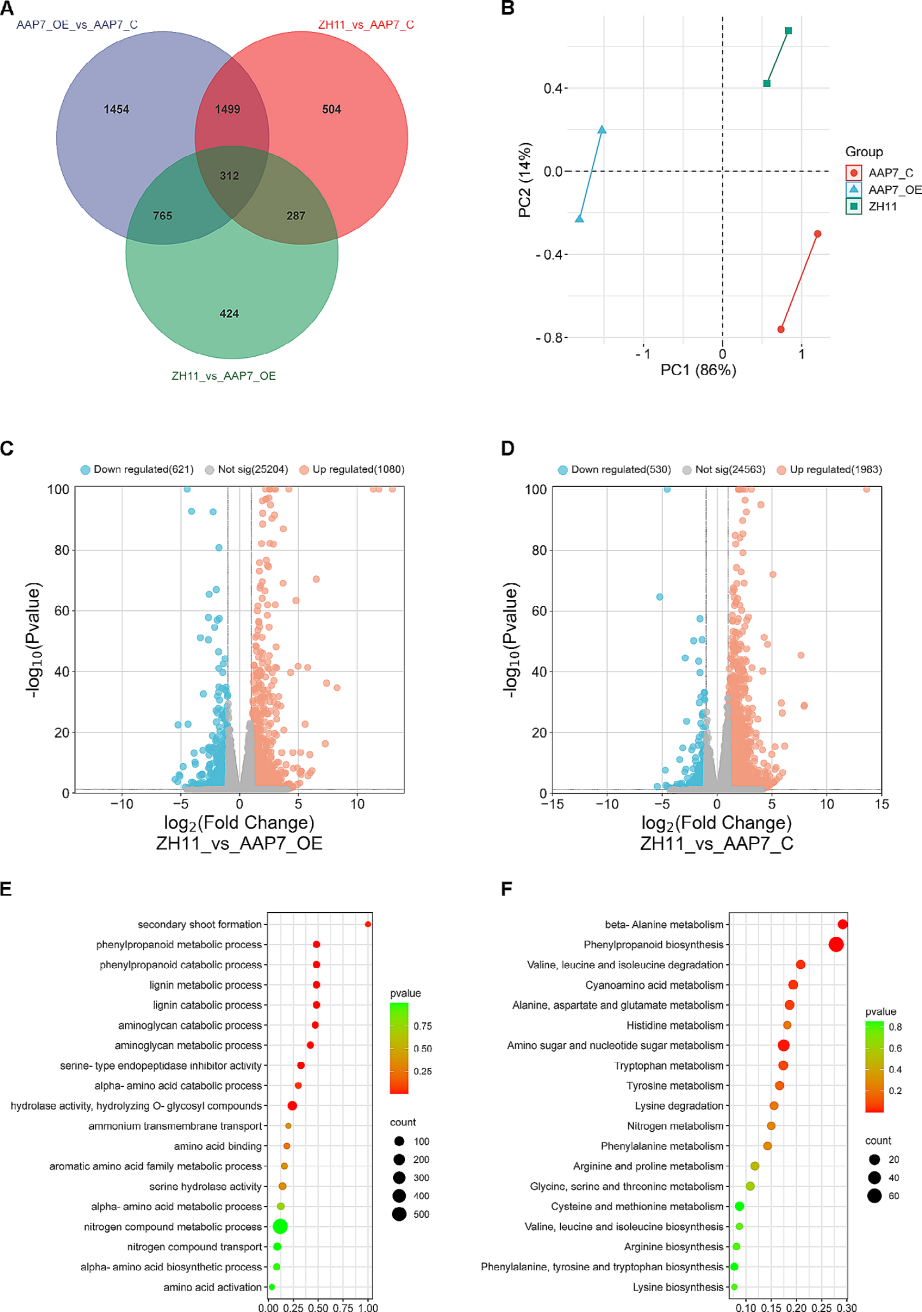
RNA-seq analysis of the axillary buds of *OsAAP7* transgenic lines. (**A**) Detection of differentially expressed genes (DEGs) in the axillary buds of *OsAAP7*-overexpressing lines (OE), *OsAAP7* CRISPR lines (C) and wild-type ZH11 (adjusted *P*-value < 0.05 and fold change > 2). (**B**) Factor map of the PCA performed on 6 samples. Following PCA analysis of the 6 samples, three clusters were identified corresponding to *OsAAP7* C lines (cluster 1, red), *OsAAP7* OE lines (cluster 2, blue), and ZH11 (cluster 3, green). (**C**-**D**) Volcano plot of ZH11 compared to *OsAAP7* OE and C lines. Orange colors represent up-regulated genes and blue colors represent down-regulated genes. (**E**-**F**) Axillary buds of *OsAAP7* transgenic lines by GO and KEGG analysis of intersections network between *OsAAP7* OE lines and C lines

To investigate the regulatory role of *OsAAP7* in axillary bud outgrowth, RNA-seq analysis was conducted on control and *OsAAP7* transgenic lines. A total of 312 differentially expressed genes (DEGs) were identified among ZH11, OE, and C lines (Fig. 7A). The reliability of the data was demonstrated among various samples of control and transgenic lines using principal component analysis (PCA) (Fig. 7B). Volcano plot analysis revealed 1086 up-regulated and 621 down-regulated genes between ZH11 and OE lines, as well as 1983 up-regulated and 530 down-regulated genes between ZH11 and C lines (Fig. 7C, D). GO and KEGG enrichment analyses of DEGs involved in the bud growth of OE and C lines of *OsAAP7* were performed. The results indicated that DEGs in transgenic lines were predominantly enriched in secondary shoot formation, phenylpropanoid, lignin, and N compound processes through GO analysis (Fig. 7E) and in beta-alanine metabolism, phenylpropanoid biosynthesis, amino acid degradation, cyanoamino acid metabolism, and phenylpropanoid biosynthesis through KEGG analysis (Fig. 7F). Additionally, GO enrichment revealed that DEGs in ZH11 and OE lines were mainly associated with amino sugar and aminoglycan processes (Supplementary Fig. S16A), while those in ZH11 and C lines were primarily related to aminoglycan, alpha-amino acid, and phenylpropanoid processes (Supplementary Fig. S16B). For the KEGG enrichment analysis, DEGs in ZH11 and OE lines exhibited enrichment mainly in phenylpropanoid biosynthesis, and beta-alanine, amino acid and N metabolisms (Supplementary Fig. S16C). Conversely, DEGs in ZH11 and C lines showed enrichment mainly in N, cyanoamino acid, starch, sucrose, and phenylalanine metabolisms (Supplementary Fig. S16D). These findings suggest that alterations in the expression of genes associated with pathways such as N metabolism, phenylpropanoid biosynthesis, and phytohormone regulation are crucial for modulating axillary bud outgrowth through changes in *OsAAP7* expression levels (Supplementary Fig. S16).

**Fig. 8:**
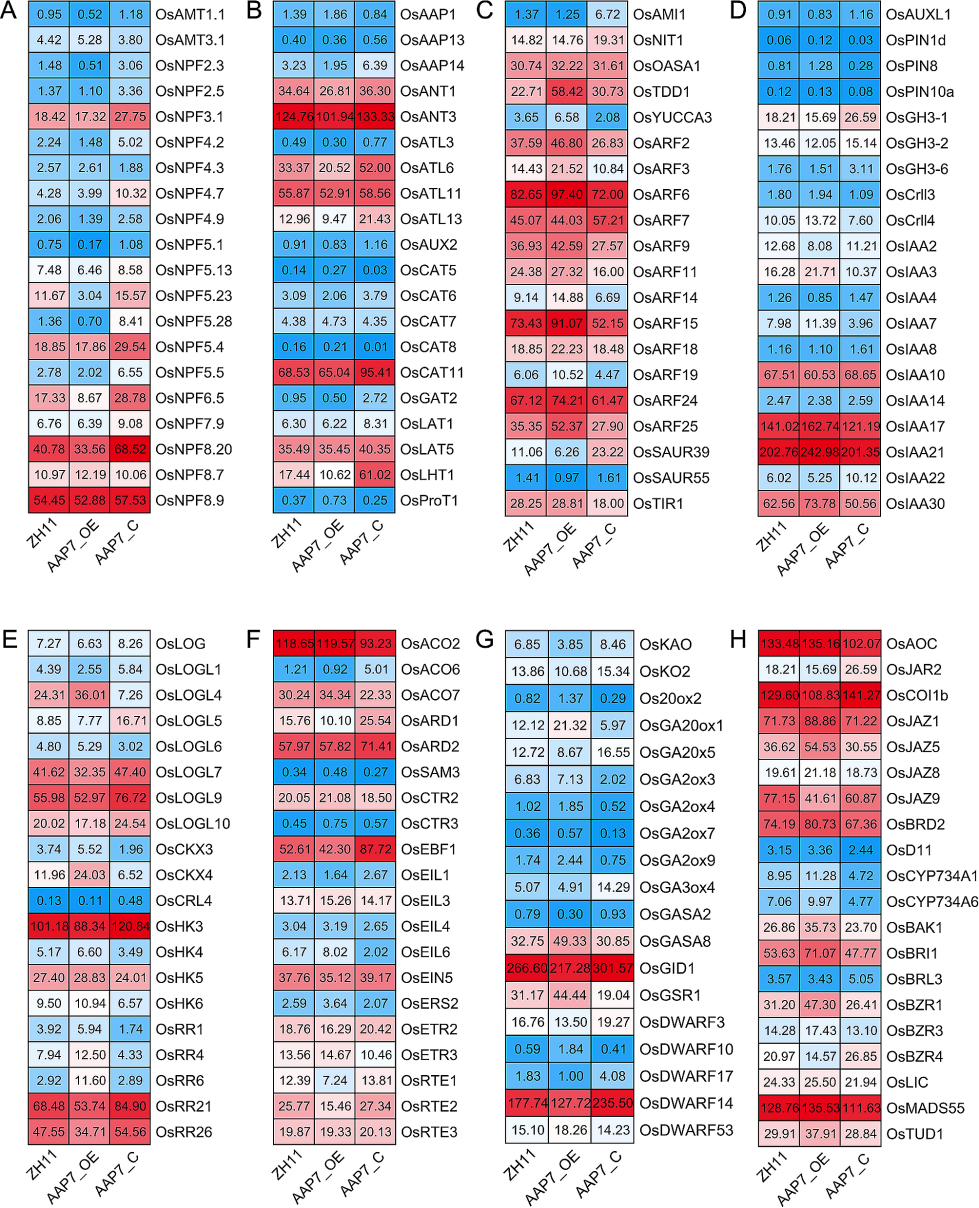
The expression pattern of DEGs participated in ammonium and nitrate transporter, amino acid transporter and phytohormone pathways in rice axillary buds of *OsAAP7* transgenic lines. (**A**-**H**) Heatmaps display up-regulated and down-regulated genes. Red and blue indicate higher and lower expression of gene

The expression patterns of DEGs related to N and phytohormone pathways in buds were analyzed using heatmaps (Fig. 8). In the mutant lines of *OsAAP7*, there was a significantly higher expression of genes encoding ammonium transporters such as *OsAMT1.1* and nitrate transporters like *OsNPF3.1*, *OsNPF6.5*, and *OsNPF8.20* compared to the ZH11 wild type, while their expression was lower in the OE lines (Fig. 8A). Similarly, the expression of amino acid transporter genes like *OsAAP14*, *OsANT1*, *OsANT3*, and *OsLHT1* was significantly higher in the mutant lines of *OsAAP7* compared to ZH11, but lower than in the OE lines (Fig. 8B). Additionally, the signaling pathways of various plant hormones, including auxin (Fig. 8C, D), cytokinin (Fig. 8E), ethylene (Fig. 8F), gibberellin and strigolactone (Fig. 8G), jasmonic acid, abscisic acid, and brassinolide (Fig. 8H) were analyzed. In the auxin pathway, genes such as *OsIAA17*, *OsIAA21*, and *OsIAA30* showed significant up-regulated in the OE lines compared to ZH11, while their expression was lower in the C lines (Fig. 8C, D). Moreover, in the cytokinin pathway, genes encoding cell division kinase activation enzymes like *OsLOG* and *OsLOGL10* were significantly up-regulated in the C lines compared to ZH11, with the opposite trend observed in the OE lines (Fig. 8E). Furthermore, genes such as *OsCKX4* [39] and other cell division kinase oxidase exhibited higher expression levels in the OE lines compared to ZH11, while their expression was lower in the C lines (Fig. 8E). The above results collectively suggested that the elongation of rice axillary buds in *OsAAP7* altered expression lines is regulated by changes in the expression of genes involved in N and various phytohormone pathways.

## Discussion

### Knockout of *OsAAP7* had the potential to increase yield by triggering tillering in rice

*Indica* and *japonica* are two key subspecies in rice, differing significantly in their biology and yield [40]. This study revealed 10 SNP sites variations in the promoter sequences of *OsAAP7* across 521 wild rice varieties (Fig. 1A). Additionally, haplotypes (hap1 and hap2) clearly distinguished between *indica* and *japonica* (Fig. 1A), suggesting natural selection of the amino acid transporter *OsAAP7* during rice evolution. Moreover, our research demonstrated that the expression level of *OsAAP7* negatively regulates rice tillering and yield (Figs. 1 and 4). To date, only two AAP members, *OsAAP3* and *OsAAP5*, have been reported to negatively regulate rice tillering [19, 21]. On the contrary, rice tillering and yield are positively regulated by other amino acid permeases, including *OsAAP1*, *OsAAP4*, *OsAAP14* and *OsAAP15* [18, 20, 23, 24]. This may be due to *OsAAP7*, *OsAAP3* and *OsAAP5* undergoing similar natural selection processes and performing analogous functions in rice. Crucially, knockout of *OsAAP7* has the potential to increase yield by stimulating tillering in rice (Fig. 4), indicating that *OsAAP7* offers a novel genetic resource for high-yield breeding application by CRISPR/Cas9 technology.

### OsAAP7 protein transported phenylalanine, lysine and arginine in rice to inhibit outgrowth of axillary bud

In this study, we demonstrated the localization of OsAAP7 protein on the ER membrane (Supplementary Fig. S2), contrasting with plasma membrane localization of OsAAP1 and OsAAP5 [18, 21]. This variance indicates distinct membrane localization among rice amino acid transporters. Then, we observed heightened expression of *OsAAP7* under treatment with basic and neutral amino acids through GUS staining and qRT-PCR (Supplementary Fig. S1). Our findings also revealed the transport of basic amino acids Lys and Arg, as well as neutral amino acid Phe, by *OsAAP7* transgenic seedlings in FITC-labeled amino acid uptake experiment (Supplementary Fig. S9). Moreover, yeast complementation experiment validated the ability of OsAAP7 protein to transport Lys and Phe (Fig. 3). Additionally, it showed that the concentrations of these amino acids Phe, Lys and Arg were significantly increased in the *OsAAP7* OE lines compared to those in ZH11 by HPLC determination (Fig. 5), indicating that these amino acids can be facilitated by OsAAP7 transporter. Previous reports have implicated AAP members such as OsAAP3 and OsAAP5 in the transport of Arg and Lys [19, 21]. Furthermore, other AAP transporters like OsAAP1, OsAAP4 and OsAAP15 primarily mediate the transport of neutral amino acids Tyr, Val or Pro in rice [18, 20, 24]. These results suggest that the accumulation of basic amino acids particularly suppresses the outgrowth of axillary buds in rice, and OsAAP3, OsAAP5 and OsAAP7 may cooperatively controls axillary bud outgrowth primarily through the transport of Arg and Lys. Importantly, amino acid transporter OsAAP7 also facilitates the transport of the aromatic amino acid Phe (Fig. 3).

### *OsAAP7* negatively regulated axillary bud growth by coordinating N and phytohormone pathways

Tillering directly influences rice yield [41] and results from the elongation of axillary buds, which are influenced by environmental cues and internal factors [42, 43]. Our result indicated that overexpression of *OsAAP7* inhibited the expression of transporters *OsNPF6.5*, *OsNPF8.20*, *OsAAP14* and *OsLHT1* in the N pathway (Fig. 8A, B). These transporters positively impact tillering in rice [23, 44–46], indicating that altered expression of *OsAAP7* controls axillary bud outgrowth by affecting transporter expression in the N pathway. In the plant hormone pathway, auxin-related genes *OsIAA17*, *OsIAA21* and *OsIAA30* [47–49] are significantly upregulated in OE lines of *OsAAP7* compared to ZH11, while the opposite pattern is showed in C lines (Fig. 8C, D). Besides, cell division kinase activator genes *OsLOG* and *OsLOG10* are notably upregulated in C lines of *OsAAP7* compared to ZH11 (Fig. 8E), suggesting that altered *OsAAP7* expression regulates axillary bud outgrowth by influencing auxin and cytokinin pathways simultaneously. This differs slightly from amino acid transporter *OsAAP5*, whose expression level only affects the cytokinin pathway in regulating bud outgrowth [21]. Based on these results, we propose that amino acid transporter *OsAAP7* negatively affects tillering and yield by mediating the transport of basic and neutral amino acids, thereby influencing N, auxin and cytokinin pathways in rice.

## Conclusion

In this study, we indicated that the haplotypes of the promoter region in amino acid transporter *OsAAP7* were divergent between *indica* and *japonica*. Moreover, it revealed that protein of OsAAP7 mainly regulated the transport of amino acids Phe, Lys, Leu and Arg. Overexpression of *OsAAP7* inhibited the outgrowth of axillary buds, while the mutant showed the opposite result. In addition, altered expression of *OsAAP7* influenced N and hormone pathways, then negatively regulated tillering and yield in rice. Overall, our findings have significant implication for rice high-yield breeding program with gene editing technology.

### Electronic supplementary material

Below is the link to the electronic supplementary material.

Supplementary Material 1

## Data Availability

All data generated or analysed during this study are included in this published article [and its supplementary information files]. The datasets generated and/or analysed during the current study are available in the National Center for Biotechnology Information (NCBI) repository.
