# Supplementary material for: Blocking of amino acid transporter OsAAP7 promoted tillering and yield by determining basic and neutral amino acids accumulation in rice
Source: BMC Plant Biol. 2024 May 23;24:447. doi: 10.1186/s12870-024-05159-5 (PMC11112796; doi:10.1186/s12870-024-05159-5)
Supplement: Supplementary file 1 — Supplementary Material 1 [file 12870_2024_5159_MOESM1_ESM.docx]

**Supplementary Table S1.** List of the primers in this study.

| Primers used for qPCR | | |
| --- | --- | --- |
| Gene | Forward sequence (5' - 3') | Reverse sequence (5' - 3') |
| *OsActin* | CGGTGTCATGGTCGGAAT | GCTCGTTGTAGAAGGTGT |
| *OsAAP7* | ATCAAGCGGGCGAACTGC | ATCTCGATGAGGATGAGGGAGT |
| Primers for plasmid construction | | |
| Gene | Forward sequence (5' - 3') | Reverse sequence (5' - 3') |
| p*OsAAP7-GUS* | TAGGATCCGGAAGGGGATGCACAATCATGAAG | TAGAATTCCTCTGCACTTTCCACAAACACCTG |
| p*OsAAP7-HBT* | ATGGATCCATGGGGGAGAACGGTGTGGTGGCG | ATGGATCCGTAGGTGGTGGCGAACGGCTTGTA |
| p*OsAAP7*-OE | ATGGTACCATGGGGGAGAACGGTGTGGTGGCG | ATGGATCCGTAGGTGGTGGCGAACGGCTTGTA |
| *U6* | GGTACCTATGTACAGCATTACGTAGG | GGTACCGATGGTGCTTACTGTTTAG |
| *U3* | GGTACCGAGCTTGTAATTCATCCAGGTC | GAGCTCGCTGTGCCGTACGACGGTACGA |
| *OsAAP7-U6sg* | TGCTTCCACGTCGAGGGGCAGTTTTAGAGCTAGAAATAGCAAGTTA | TGCCCCTCGACGTGGAAGCAAACCTGAGCCTCAGCGCAGC |
| *OsAAP7-U3sg* | TGCAGAAGAGGATACCGAGGGTTTTAGAGCTAGAAATAGCAAGTTA | CCTCGGTATCCTCTTCTGCAGCCACGGATCATCTGCACAACTC |
| *OsAAP7-pDR196* | AAGAATTCATGGGGGAGAACGGTGTGGTGGCG | AACTCGAGGTAGGTGGTGGCGAACGGCTTGTA |
| Primers for OsAAP7 identification | | |
| Gene | Forward sequence (5' - 3') | Reverse sequence (5' - 3') |
| *OsAAP7-C* | TGCTCACTGTGCTGGCATTT | CACGGACCAGAAACAATCAAAA |
| *HYG* | CTTCTACACAGCCATCGGTCCAG | CGGAAGTGCTTGACATTGGGGAG |
| *HBT* | ACTCTCTCGCCACACACACCC | ACTTGTGGCCGTTTACGTCGC |

**
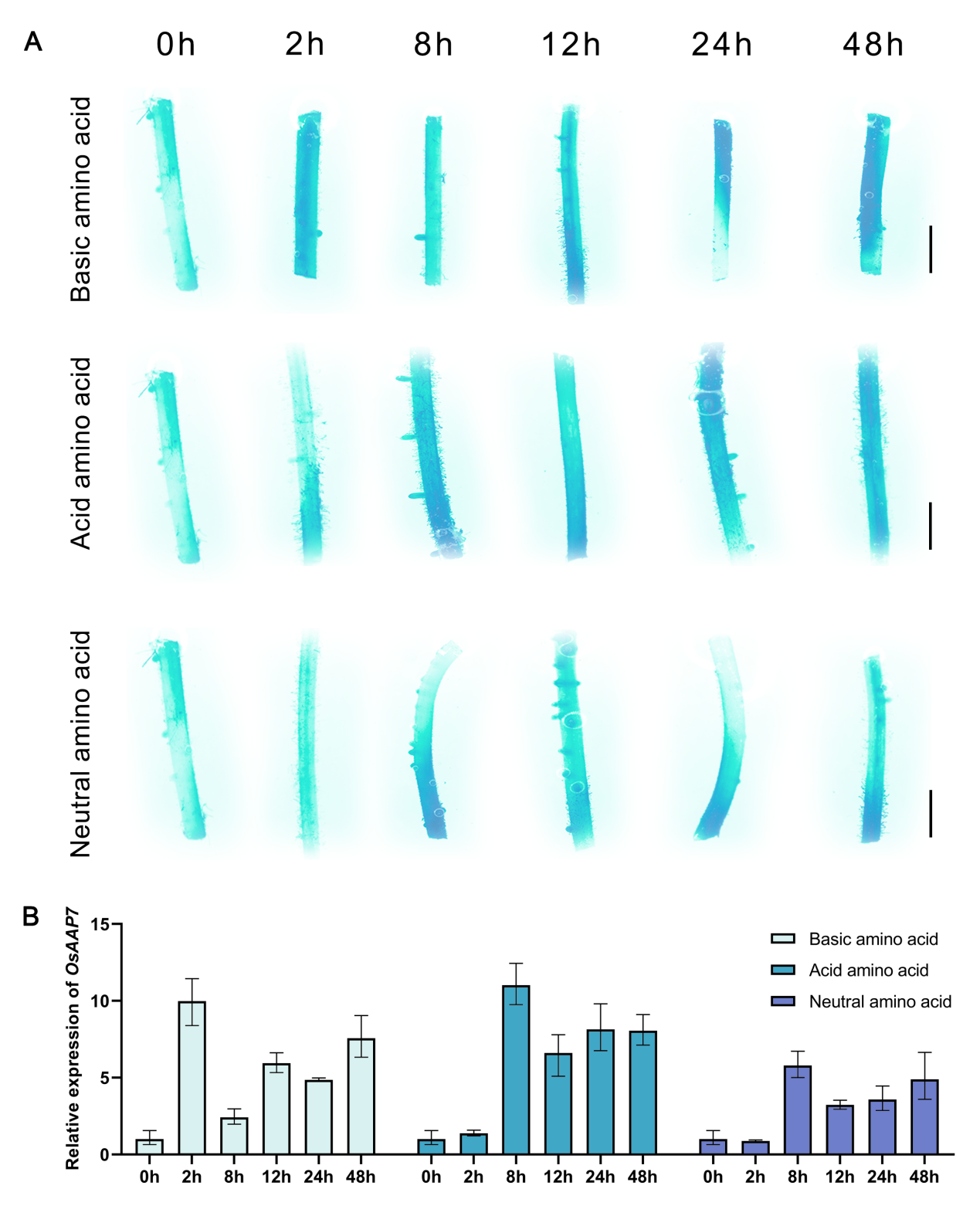
****Supplementary Fig. S1.** Analysis of GUS-stained lateral roots after different amino acid treatments of *OsAAP7* promoter-GUS transgenic seedlings. **A** Phenotypes of GUS staining of lateral roots after treating *OsAAP7* promoter-GUS transgenic seedlings with basic, acidic and neutral amino acids. Scale bars = 5 mm. **B** Relative expression of *OsAAP7* during treatment of transgenic seedlings with basic, acidic and neutral amino acids. Error bars indicate SD (n=5).

**
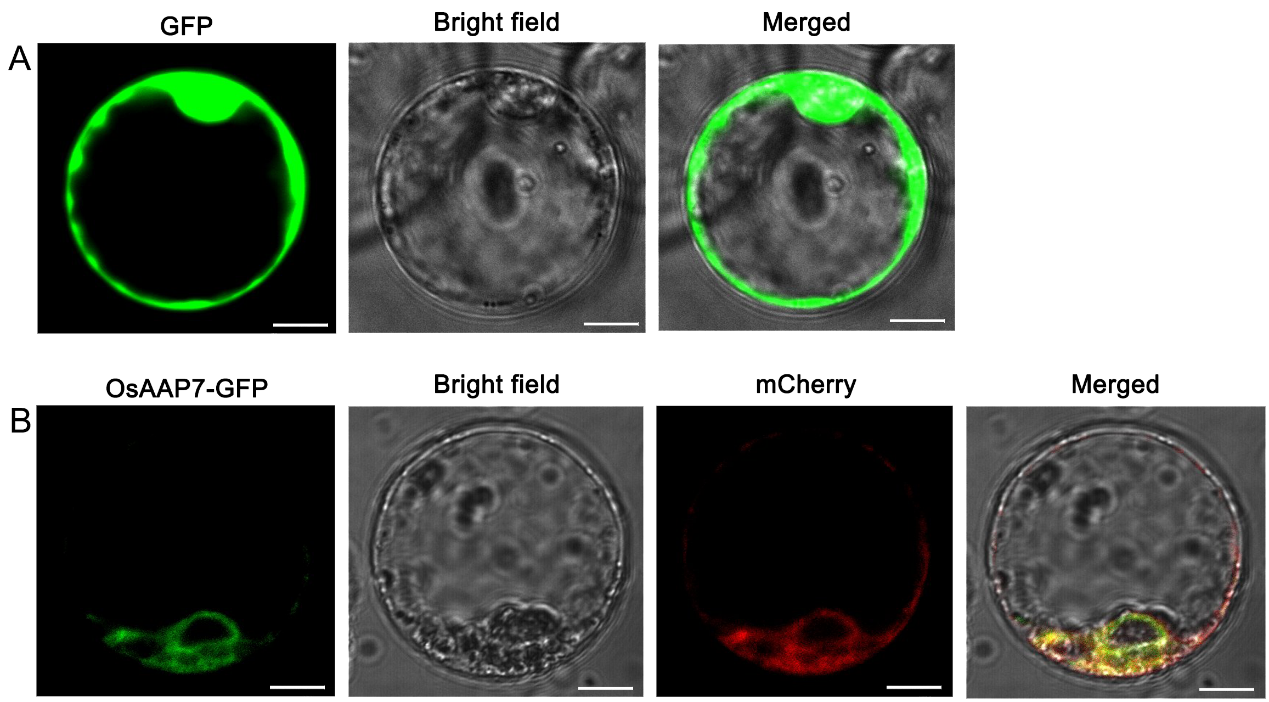
**

**Supplementary Fig. S2.** Subcellular Localization of OsAAP7. **A** Expression of free green fluorescent protein in rice protoplasts. **B** Expression of *OsAAP7*-GFP, subcellular localization and co-localization with mCherry-fused endoplasmic reticulum membrane protein GFP control. Scale bars=10 μm.


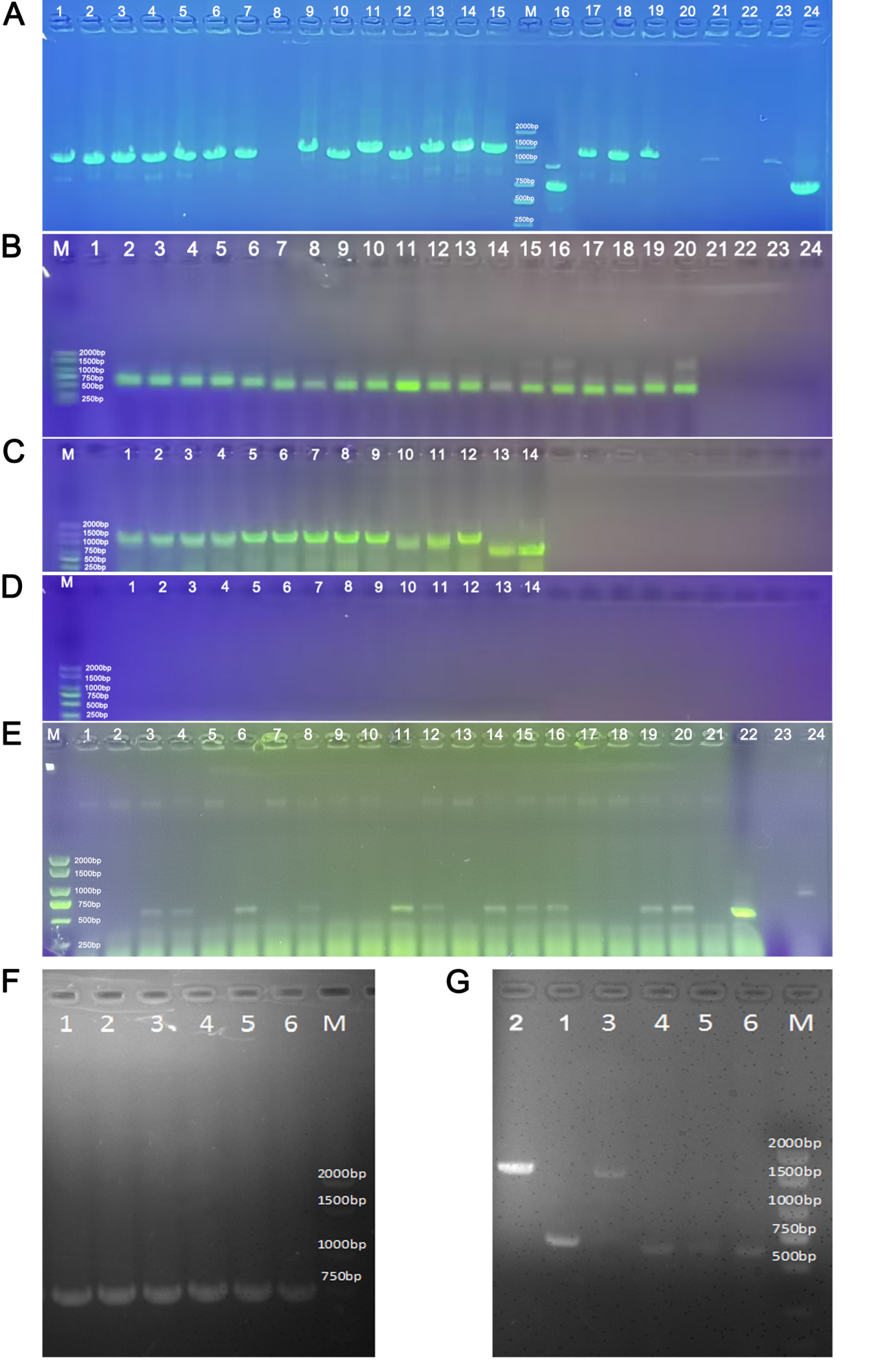


**Supplementary Fig. S3.** The identification of transgenic plants. **A** Amplification of *OsAAP7* gene coding region in 24 T0-generation mutant plants (C lines). **B** Amplification of Cas9 gene in 24 T0-generation mutant plants (C lines). **C** Amplification of *OsAAP7* gene coding region in 14 T1-generation mutant plants (C lines). **D** Amplification of Cas9 gene in 14 T2 mutant plants (C lines). **E** Amplification of *hygromycin-*gene in 24 T0-generation overexpressed plants (OE lines). **F** Amplification of *hygromycin-*gene in 6 T1-generation overexpressed plants (OE lines). **G** *OsAAP7* gene coding region in T2 mutant plants (1-3 correspond to C1-C3 lines) and hygromycin gene in overexpressed plants (1-3 correspond to OE1-OE3 lines).


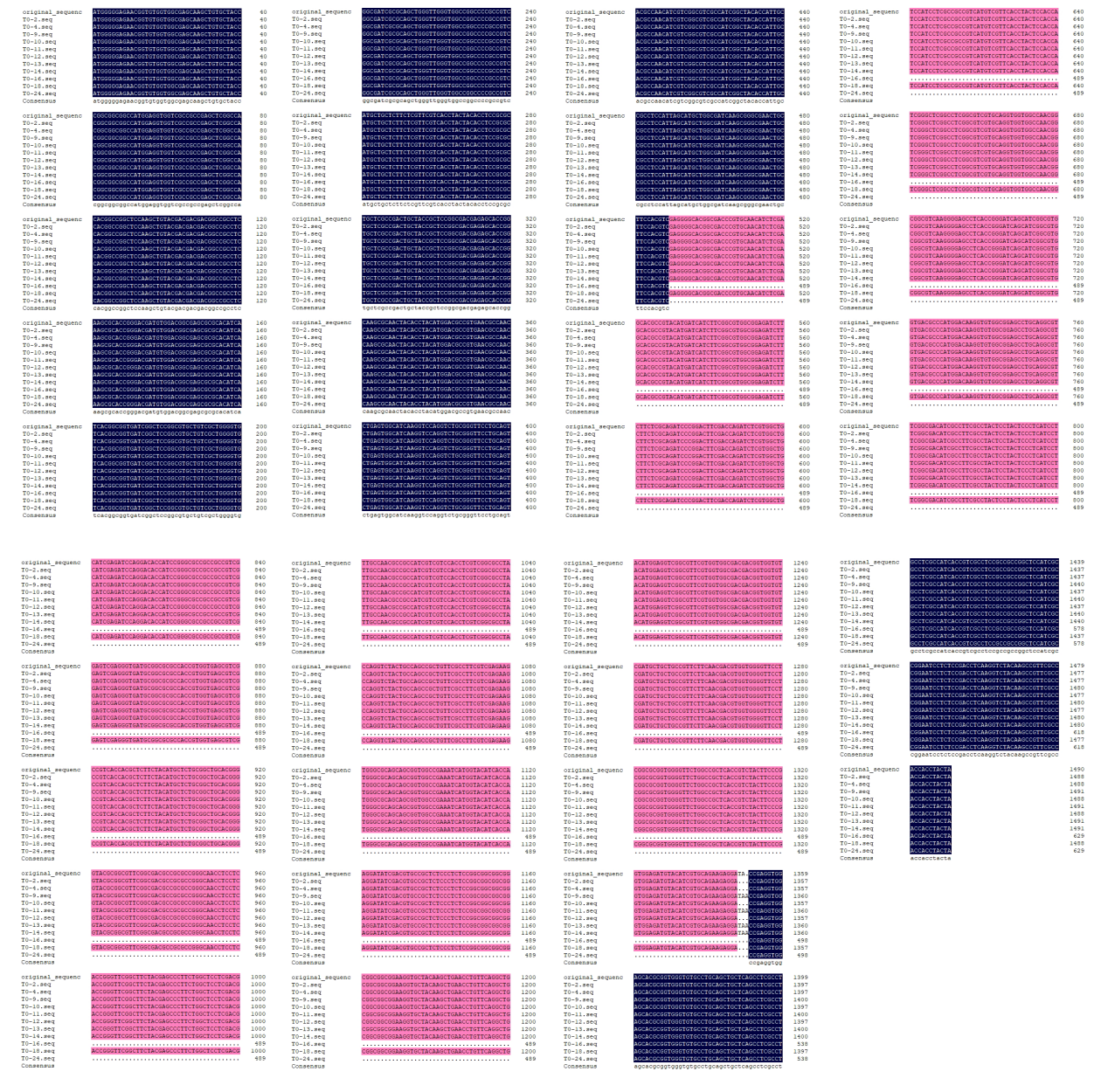


**Supplementary Fig. S4.** Sequencing results of T0-generation mutants.


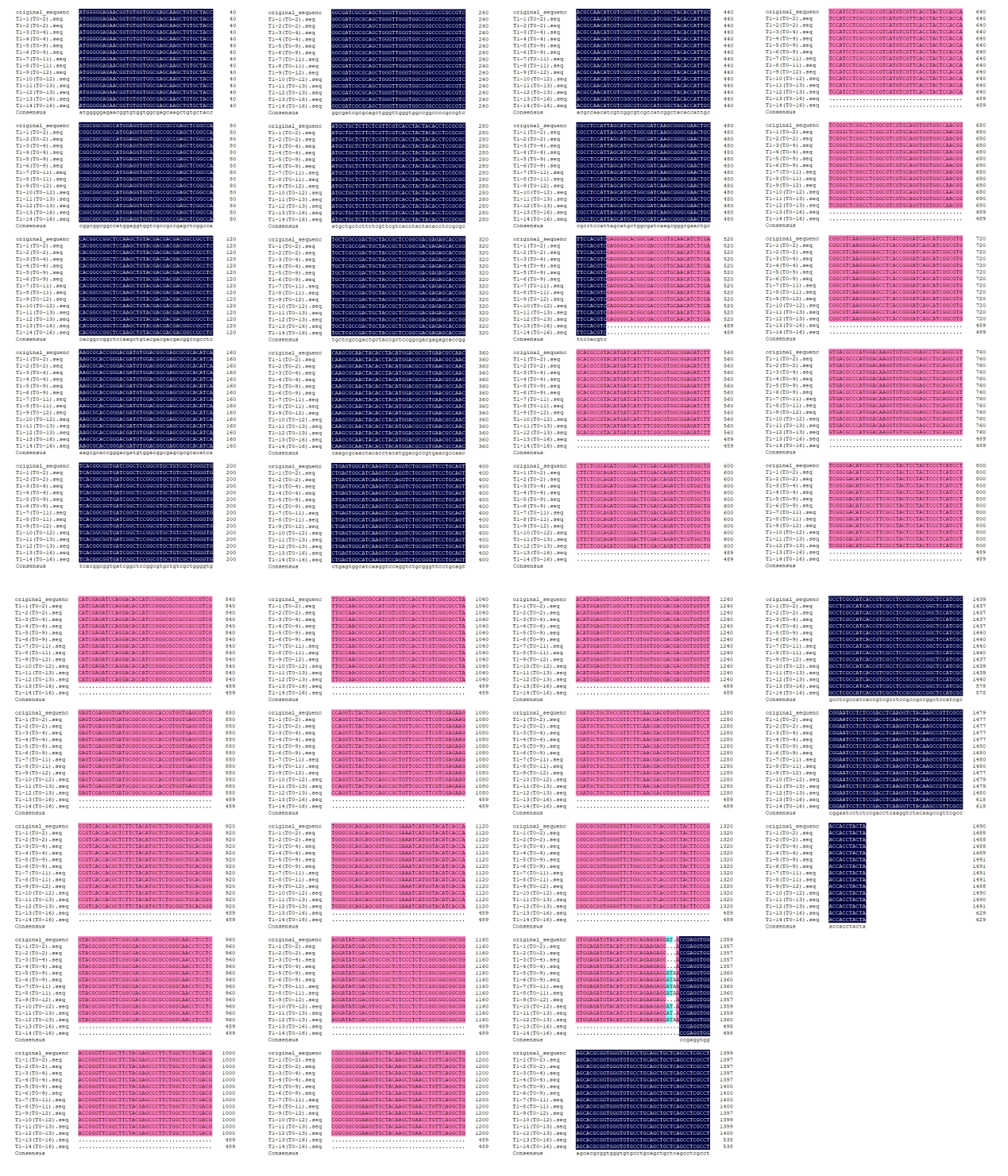


**Supplementary Fig. S5.** Sequencing results of T1-generation mutants.


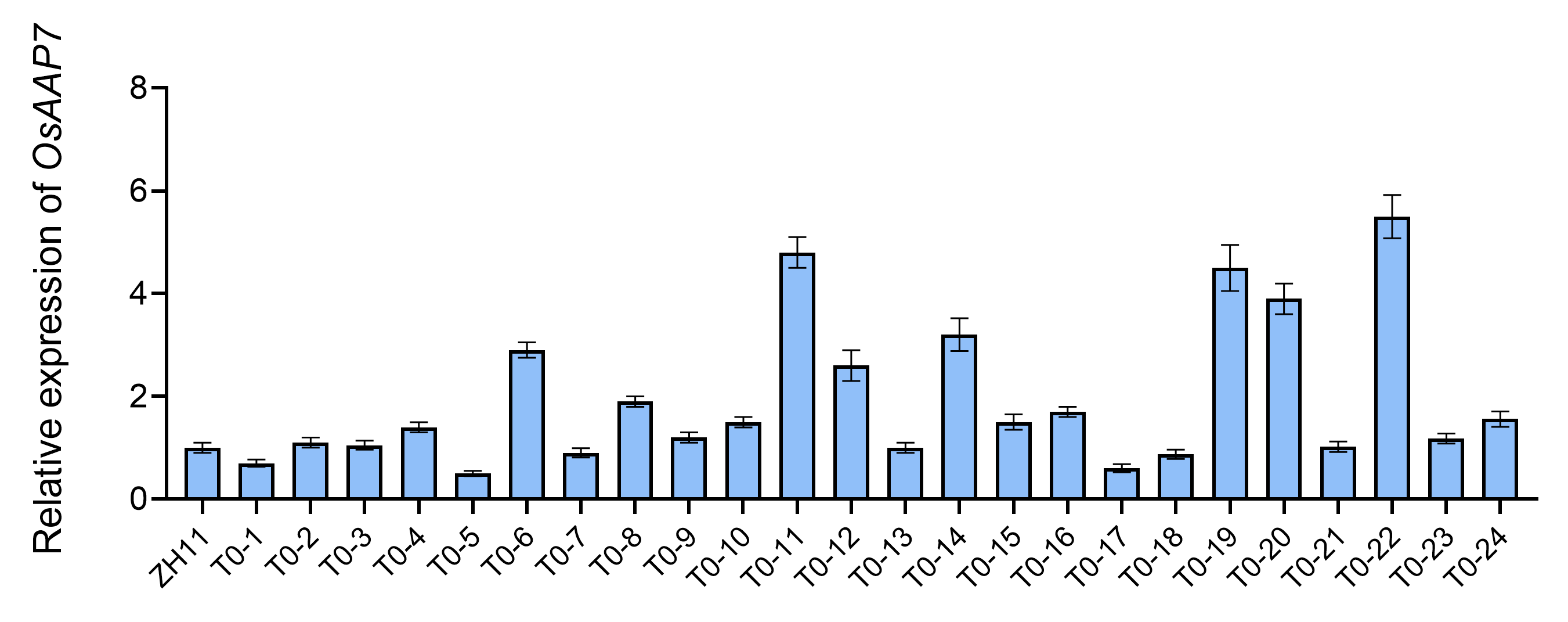


**Supplementary Fig. S6.** Identification of *OsAAP7* gene expression in leave blade of T0-generation overexpressed plants (OE lines).


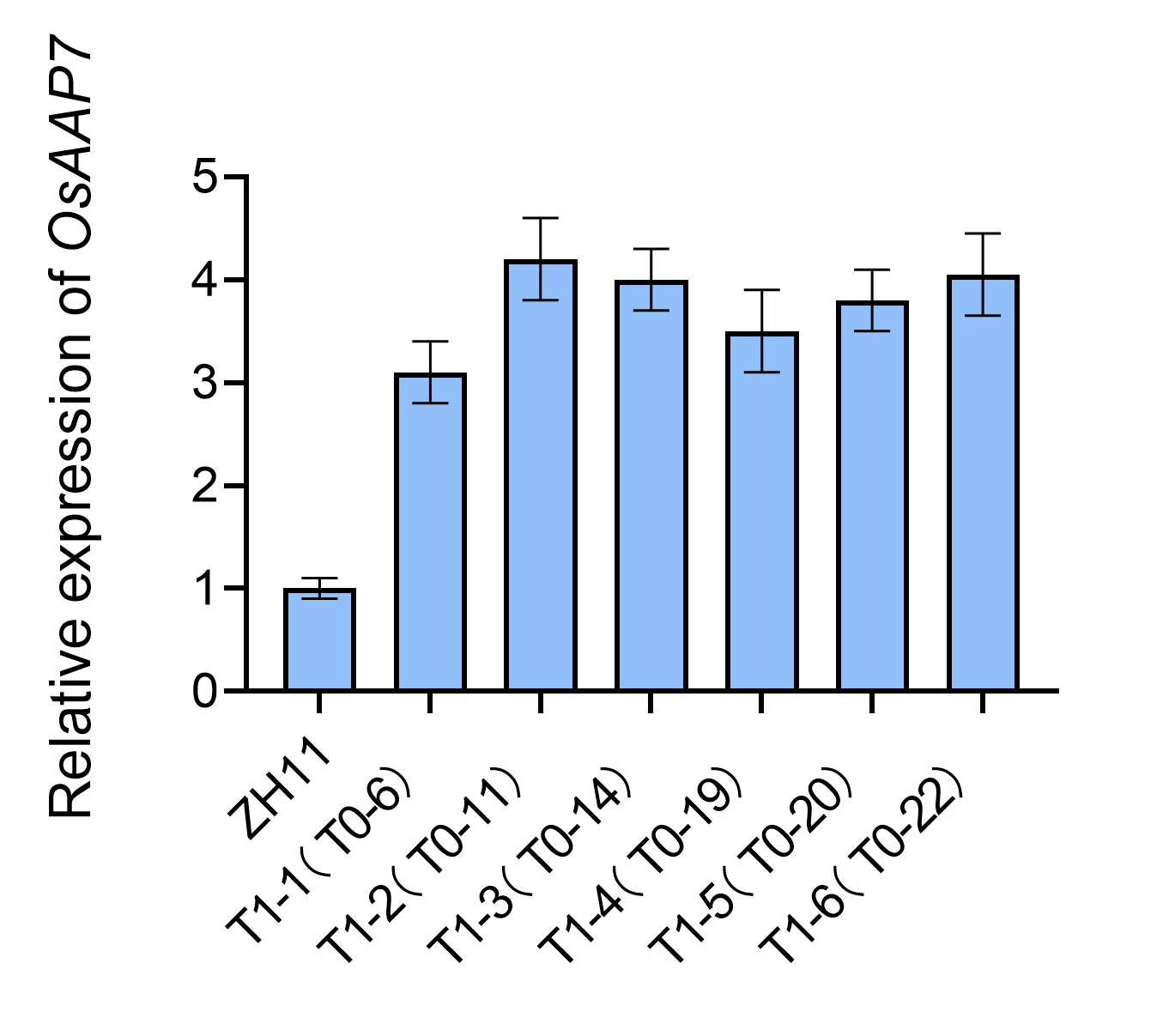


**Supplementary Fig. S7.** Identification of *OsAAP7* gene expression in leave blade of T1-generation overexpressed plants (OE lines).


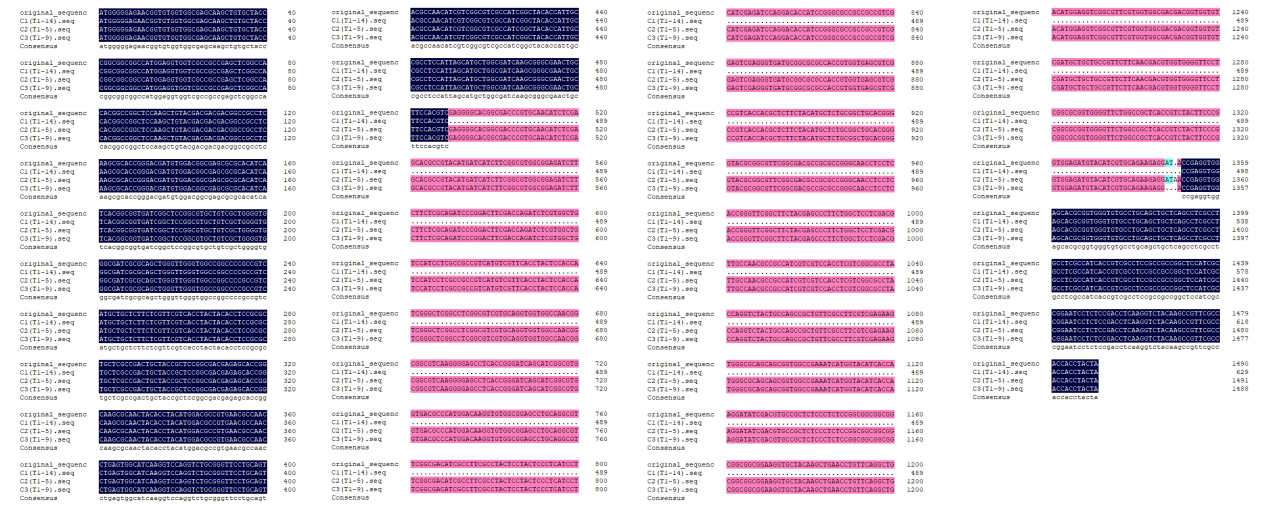


**Supplementary Fig. S8.** Sequencing results of T2-generation mutants (C1-C3).


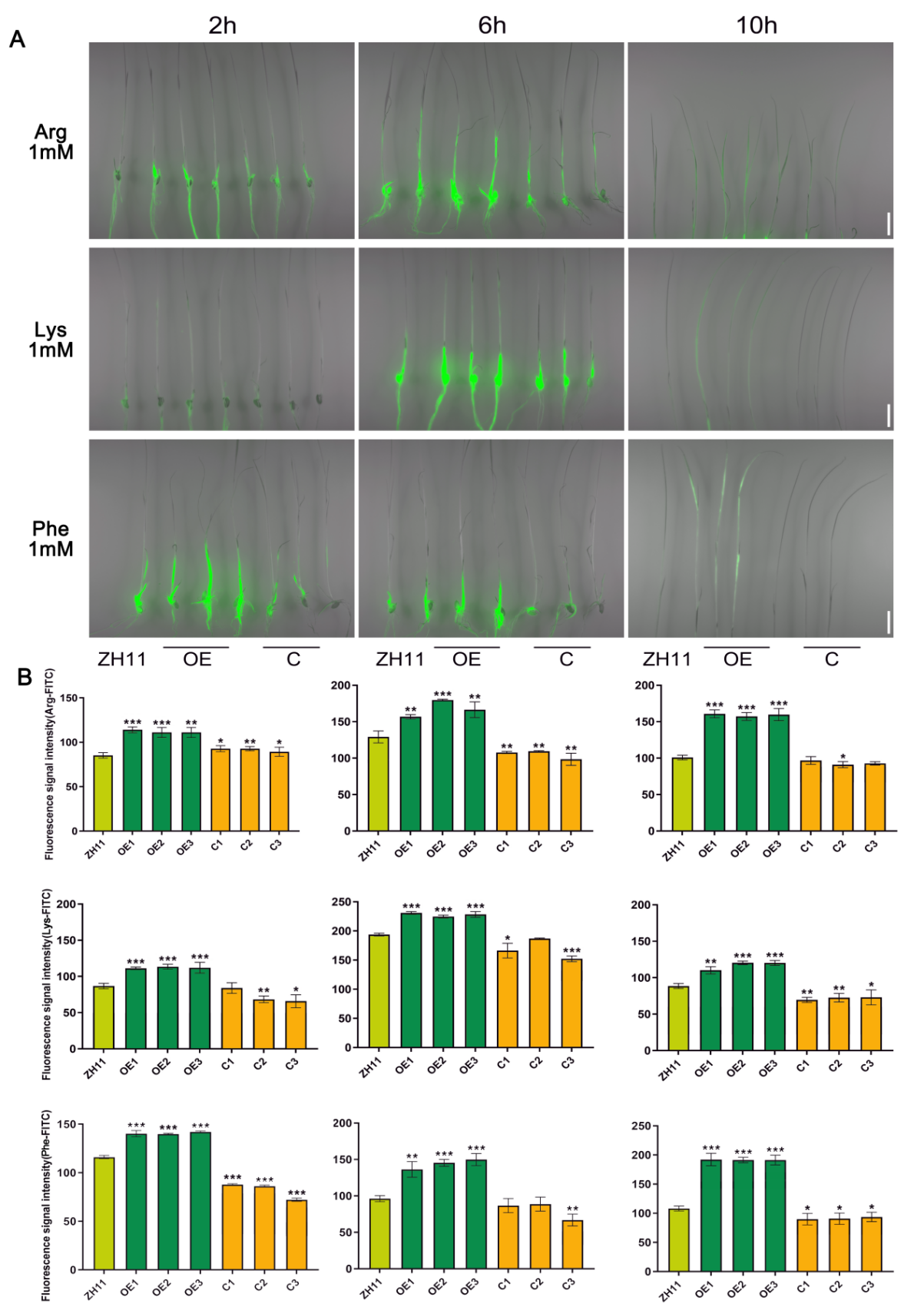


**Supplementary Fig. S9.** Fluorescence amino acid uptake was determined in seedlings of *OsAAP7* transgenic plants. **A** Phenotypes of wild-type ZH11, *OsAAP7*-overexpressing lines (OE), *OsAAP7* CRISPR lines (C) seedlings incubated with FITC-labeled amino acids Arg, Lys, and Phe for 2, 6, and 10 hours. Scale bars = 2 mm. **B** Fluorescence signal intensity was measured after incubating seedlings of *OsAAP7* transgenic plants with FITC-labeled amino acid Arg, Lys, Phe. Error bars depict the SD (n=20). *, ** and *** indicate significant differences at *P*<0.05, *P*<0.01 and *P*<0.001, respectively.

**
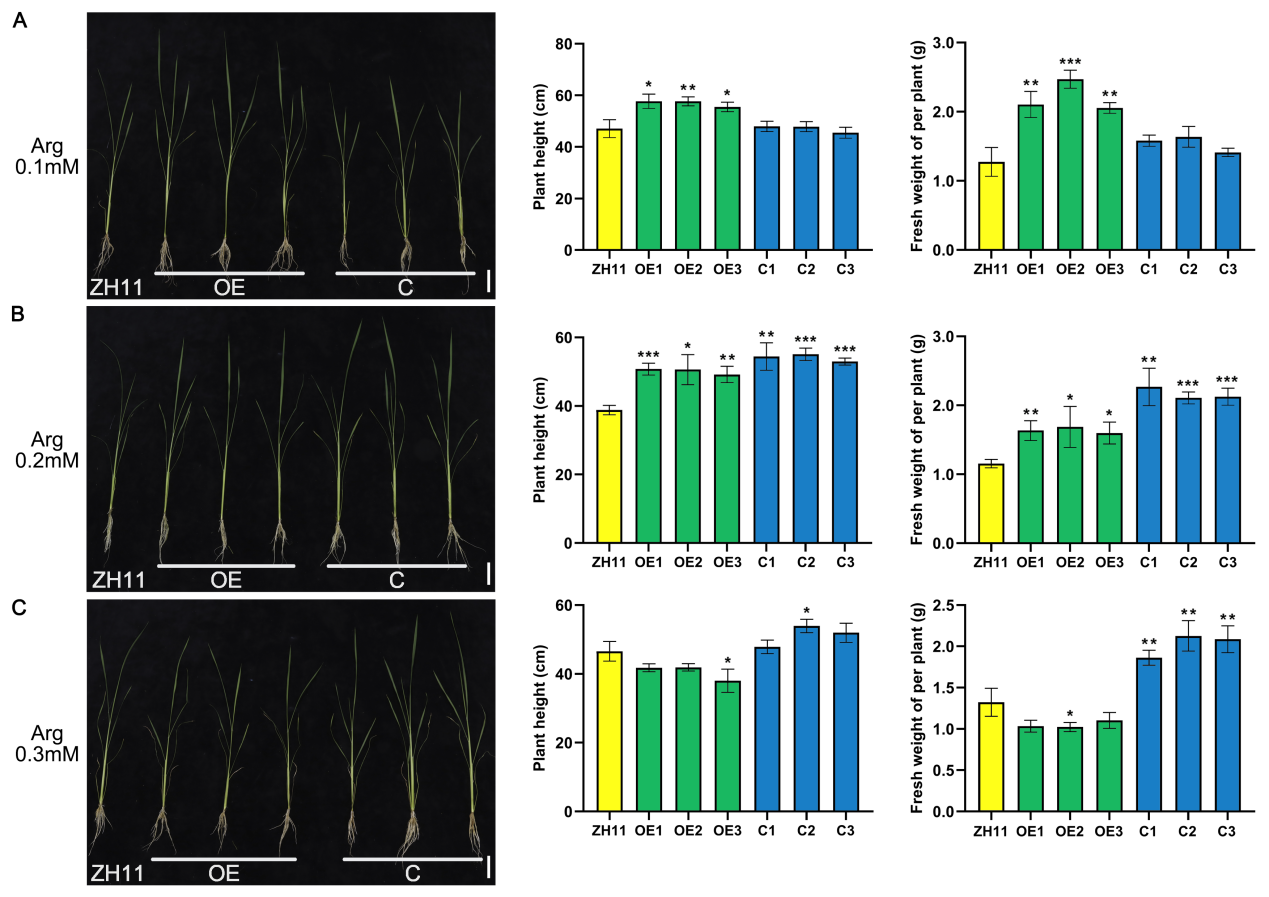
****Supplementary Fig. S10.** Seedling growth of *OsAAP7* transgenic plants at different arginine (Arg) concentrations. **A**-**C** Plant length and fresh weight of wild-type ZH11, *OsAAP7*-overexpressing lines (OE1-OE3), and *OsAAP7* CRISPR lines (C1-C3) after 35 days of incubation in the nutrient solution of 0.1 mM Arg, 0.2 mM Arg, and 0.3 mM Arg. Scale bar = 5 cm. Error bars indicate SD (n=20). *, ** and *** indicate significant differences (*P*<0.05, *P*<0.01 and *P*<0.001, respectively).

**
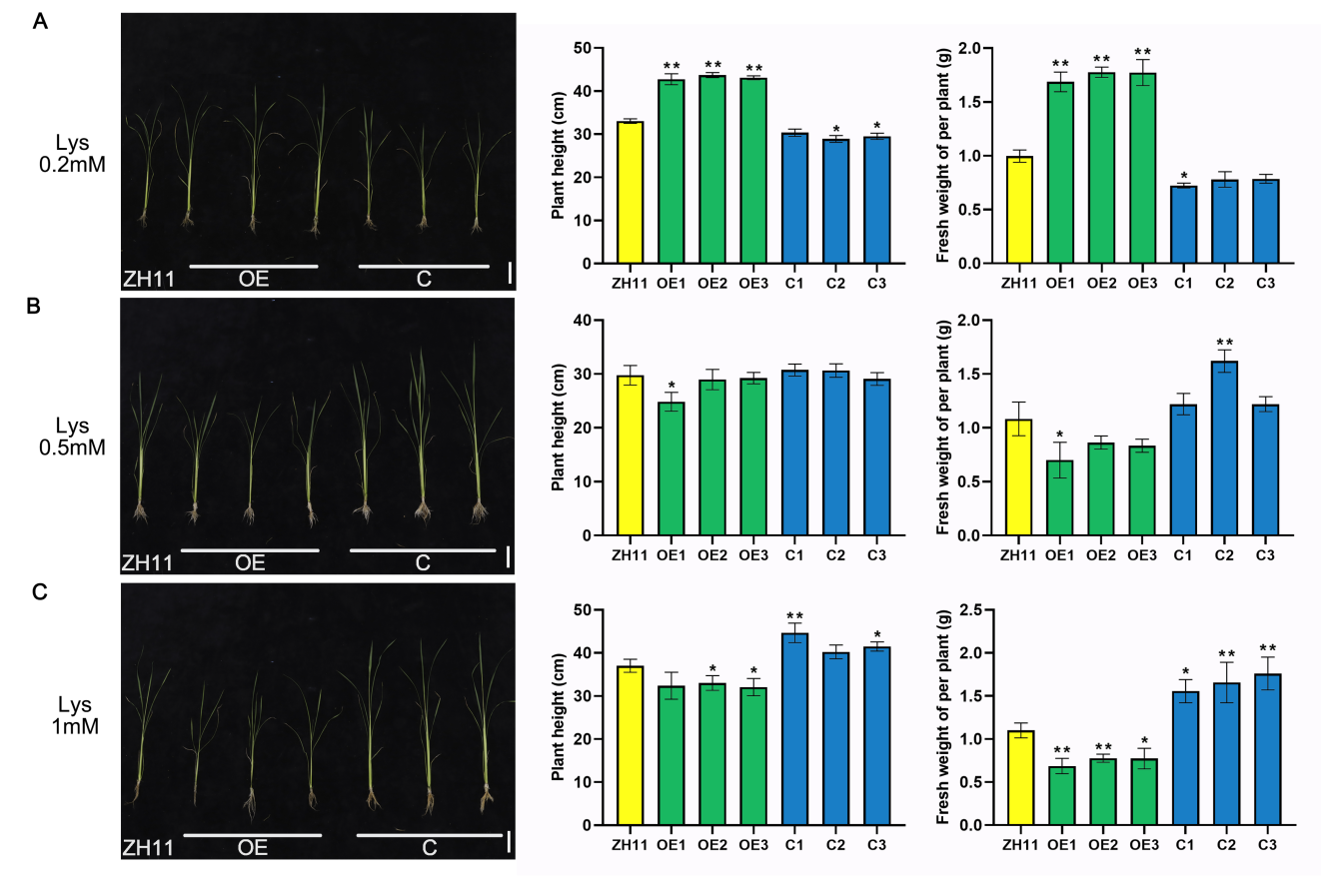
** **Supplementary Fig. S11.** Seedling growth of *OsAAP7* transgenic plants at different lysine (Lys) concentrations. **A**-**C** Plant length and fresh weight of wild-type ZH11, *OsAAP7*-overexpressing lines (OE1-OE3), and *OsAAP7* CRISPR lines (C1-C3) after 35 days of incubation in the nutrient solution of 0.5 mM Lys, 1 mM Lys, and 2 mM Lys. Scale bar = 5 cm. Error bars indicate SD (n=20). *, ** and *** indicate significant differences (*P*<0.05, *P*<0.01 and *P*<0.001, respectively).

**
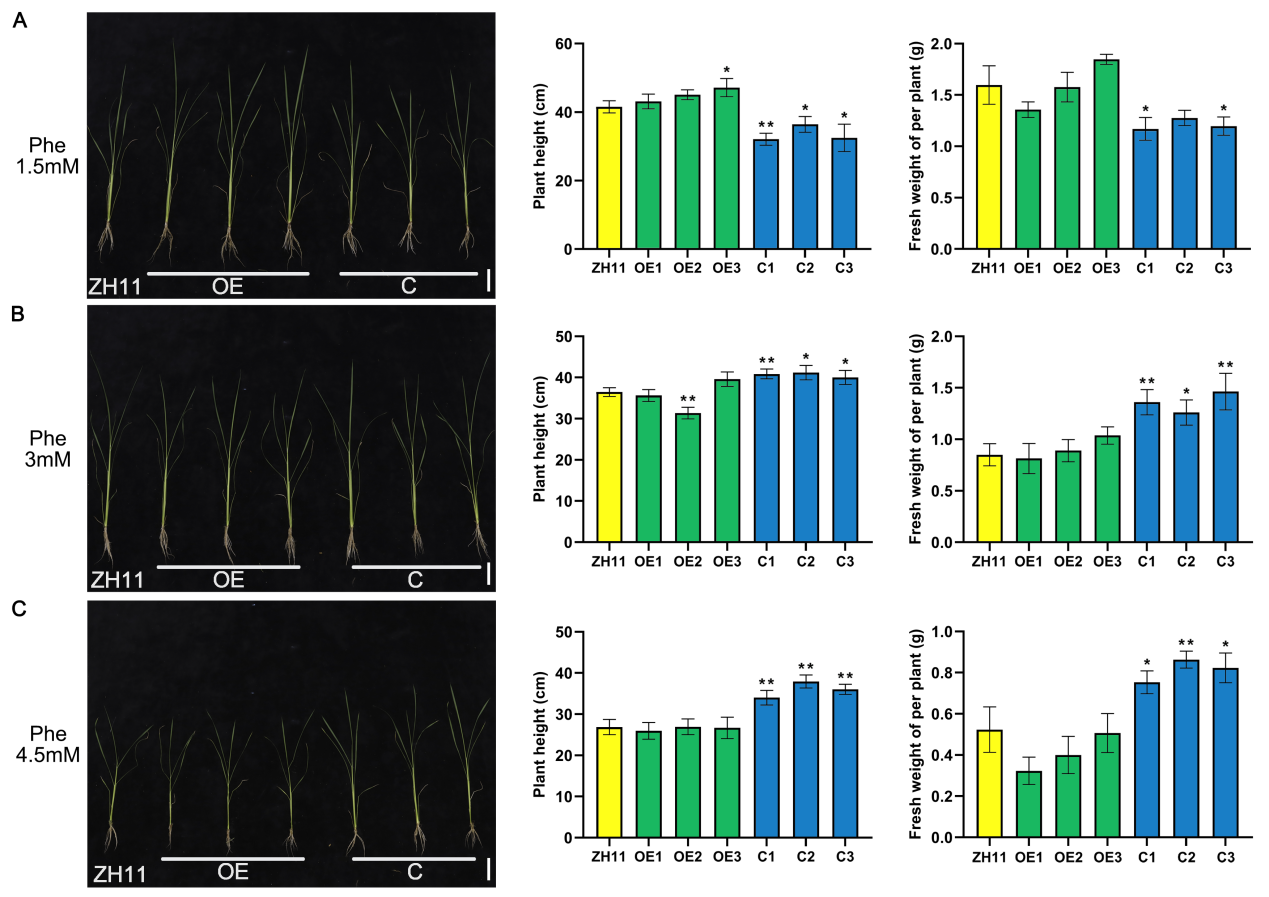
Supplementary Fig. S12.** Seedling growth of *OsAAP7* transgenic plants at different phenylalanine (Phe) concentrations. **A**-**C** Plant length and fresh weight of wild-type ZH11, *OsAAP7*-overexpressing lines (OE1-OE3), and *OsAAP7* CRISPR lines (C1-C3) after 35 days of incubation in the nutrient solution of 1.5 mM Phe, 3 mM Phe, and 4.5 mM Phe. Scale bar = 5 cm. Error bars indicate SD (n=20). *, ** and *** indicate significant differences (*P*<0.05, *P*<0.01 and *P*<0.001, respectively).

**
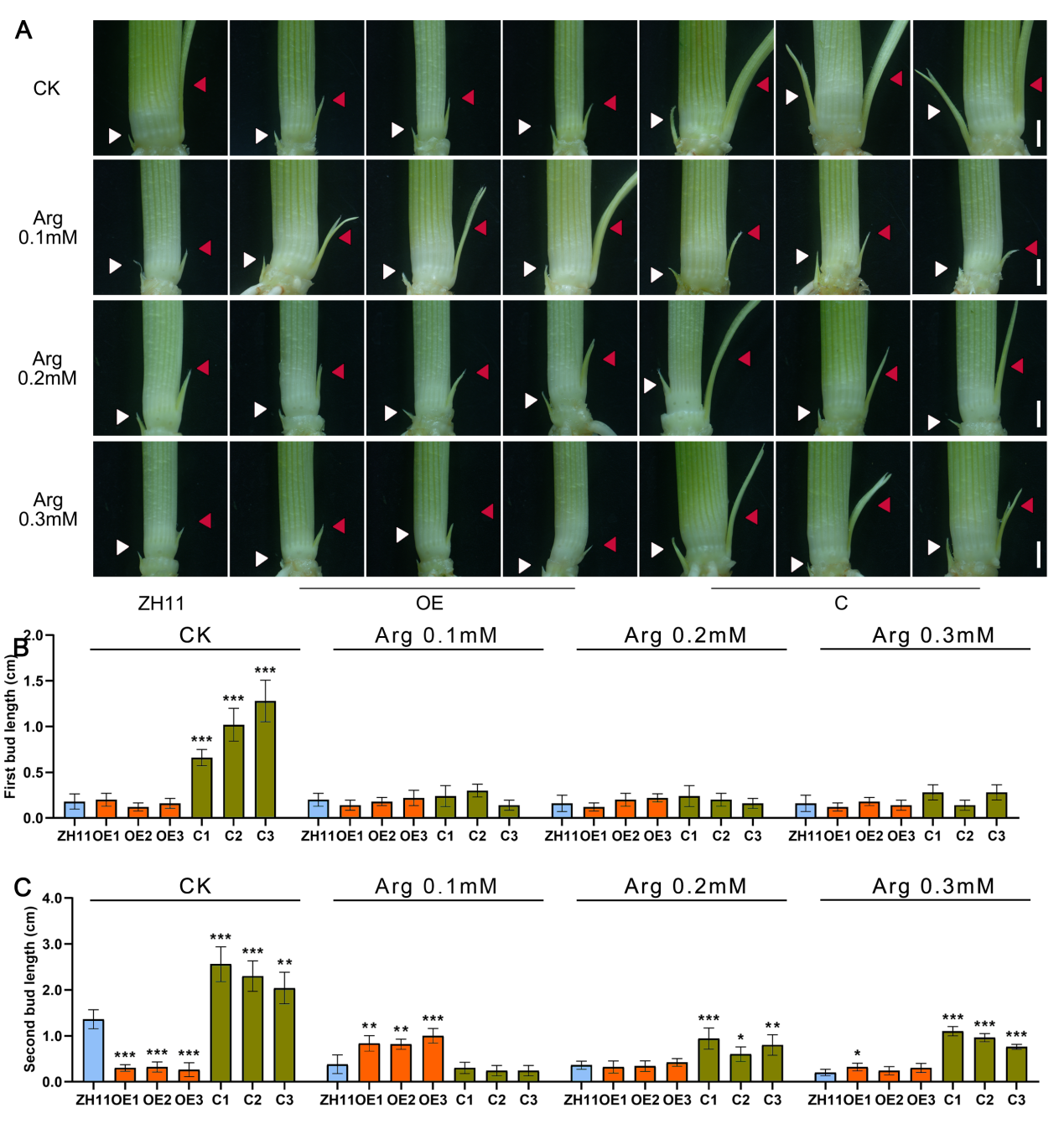
 Supplementary Fig. S13.** Growth of axillary buds of *OsAAP7* transgenic plants at different arginine (Arg) concentrations. **A** Phenotypes of axillary bud of wild-type ZH11, *OsAAP7*-overexpressing lines (OE1-OE3), *OsAAP7* CRISPR lines (C1-C3) under 0.1 mM Arg, 0.2 mM Arg, 0.3 mM Arg in nutrient solution culture when the rice plants were grown for 35 days. Scale bars = 2 mm. **B**-**C** First bud length and second bud length of wild-type ZH11, *OsAAP7*-overexpressing lines (OE1-OE3), *OsAAP7* CRISPR lines (C1-C3) under 0.1 mM Arg, 0.2 mM Arg, 0.3 mM Arg in nutrient solution culture when the rice plants were grown for 35 days. Error bars depict the SD (n=20). *, ** and *** indicate significant differences at *P*<0.05, *P*<0.01 and *P*<0.001, respectively. The white arrow represents the first axillary bud and the red arrow represents the second axillary bud.

**
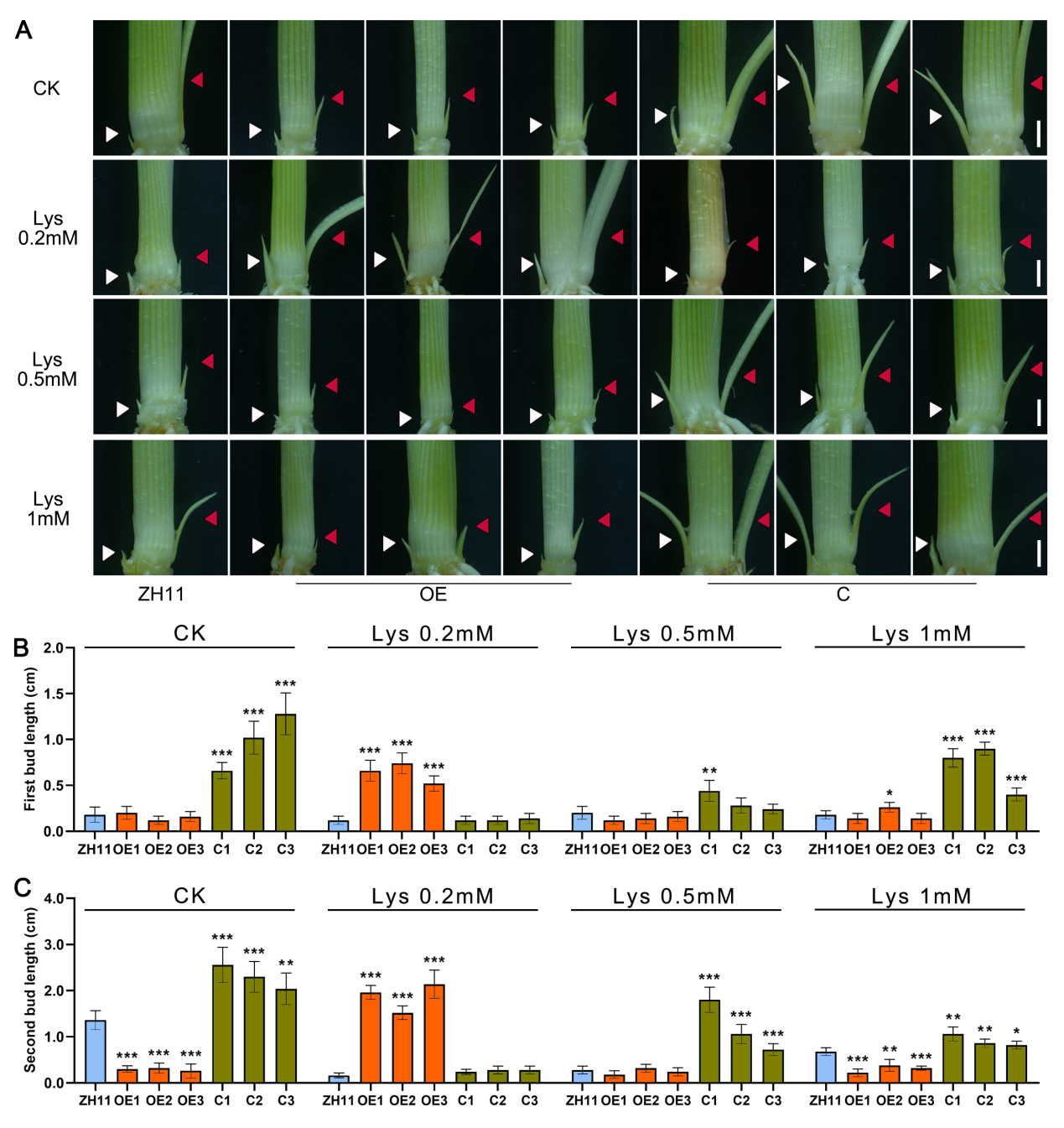
Supplementary Fig. S14.** Growth of axillary buds of *OsAAP7* transgenic plants at different lysine (Lys) concentrations. **A** Phenotypes of axillary bud of wild-type ZH11, *OsAAP7*-overexpressing lines (OE1-OE3), *OsAAP7* CRISPR lines (C1-C3) under 0.5 mM Lys, 1 mM Lys, 2 mM Lys in nutrient solution culture when the rice plants were grown for 35 days. Scale bars = 2 mm. **B**-**C** First bud length and second bud length of wild-type ZH11, *OsAAP7*-overexpressing lines (OE1-OE3), *OsAAP7* CRISPR lines (C1-C3) under 0.5 mM Lys, 1 mM Lys, 2 mM Lys in nutrient solution culture when the rice plants were grown for 35 days. Error bars depict the SD (n=20). *, ** and *** indicate significant differences at *P*<0.05, *P*<0.01 and *P*<0.001, respectively. The white arrow represents the first axillary bud and the red arrow represents the second axillary bud.

**
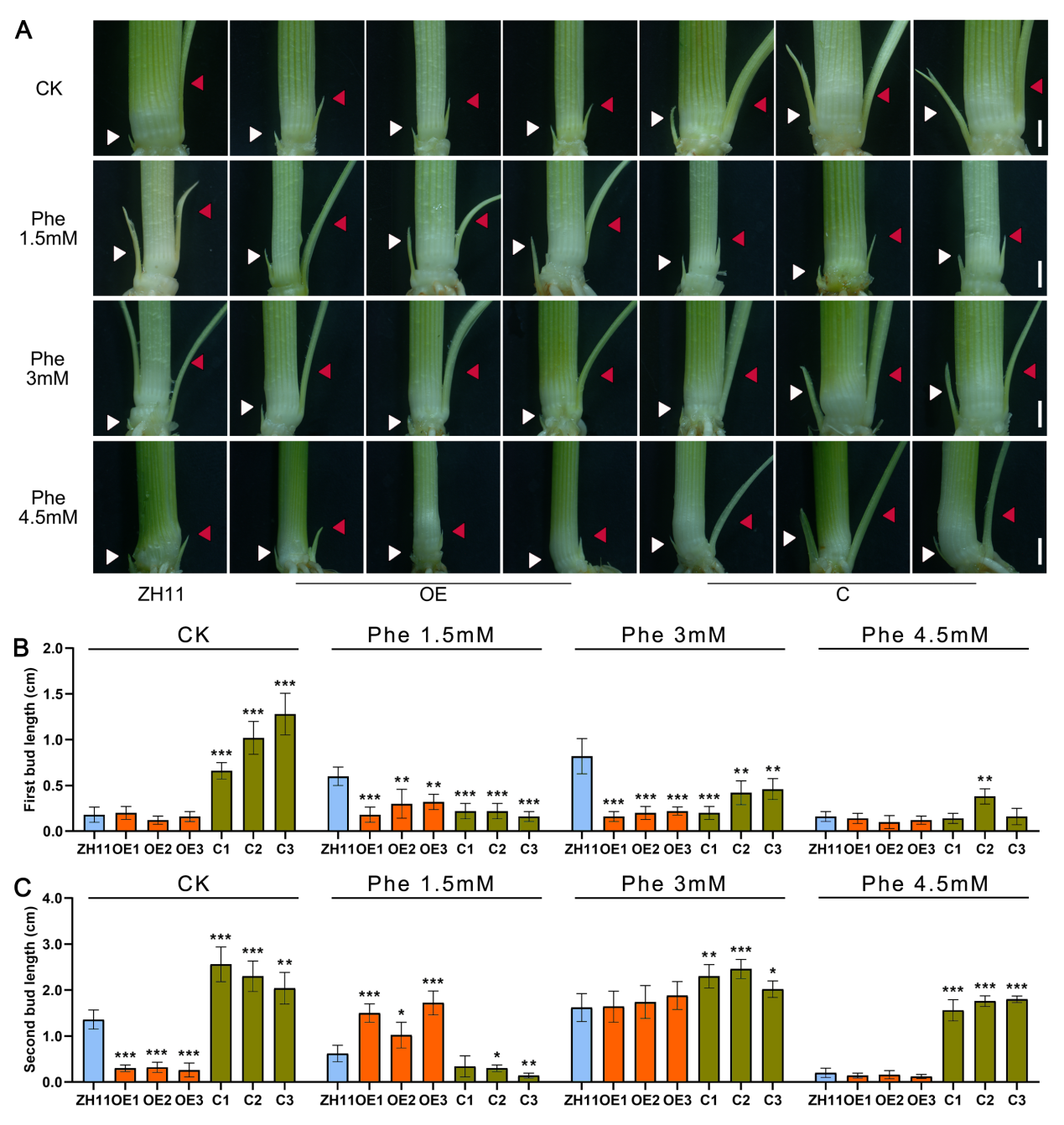
Supplementary Fig. S15.** Growth of axillary buds of *OsAAP7* transgenic plants at different phenylalanine (Phe) concentrations. **A** Phenotypes of axillary bud of wild-type ZH11, *OsAAP7*-overexpressing lines (OE1-OE3), *OsAAP7* CRISPR lines (C1-C3) under 1.5 mM Phe, 3 mM Phe, 4.5 mM Phe in nutrient solution culture when the rice plants were grown for 35 days. Scale bars = 2 mm. **B**-**C** First bud length and second bud length of wild-type ZH11, *OsAAP7*-overexpressing lines (OE1-OE3), *OsAAP7* CRISPR lines (C1-C3) under 1.5 mM Phe, 3 mM Phe, 4.5 mM Phe in nutrient solution culture when the rice plants were grown for 35 days. Error bars depict the SD (n=20). *, ** and *** indicate significant differences at *P*<0.05, *P*<0.01 and *P*<0.001, respectively. The white arrow represents the first axillary bud and the red arrow represents the second axillary bud.

**
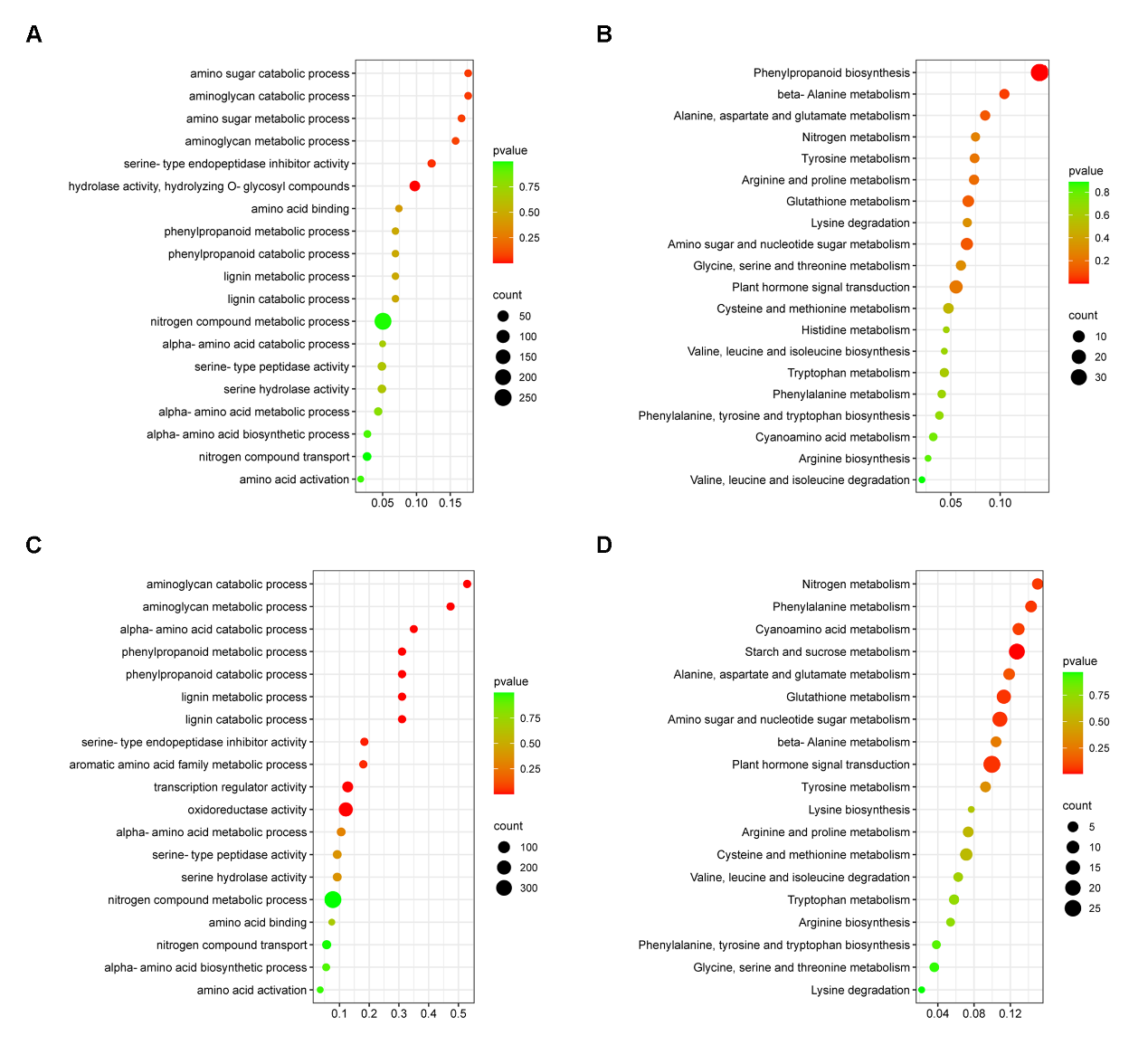
****Supplementary Fig. S16.** Transcriptome analysis of *OsAAP7* transgenic plants in the growing axillary buds. **A**-**B** Axillary buds of *OsAAP7* transgenic plants by GO and KEGG analysis of intersections network between ZH11 and *OsAAP7*-overexpressing lines. **C**-**D** Axillary buds of *OsAAP7* transgenic plants by GO and KEGG analysis of intersections network between ZH11 and *OsAAP7* CRISPR lines.
